# Association of Generalized Anxiety Disorder With Autonomic Hypersensitivity and Blunted Ventromedial Prefrontal Cortex Activity During Peripheral Adrenergic Stimulation: A Randomized Clinical Trial

**DOI:** 10.1001/jamapsychiatry.2021.4225

**Published:** 2022-02-02

**Authors:** Adam R. Teed, Justin S. Feinstein, Maria Puhl, Rachel C. Lapidus, Valerie Upshaw, Rayus T. Kuplicki, Jerzy Bodurka, Olujimi A. Ajijola, Walter H. Kaye, Wesley K. Thompson, Martin P. Paulus, Sahib S. Khalsa

**Affiliations:** 1 Laureate Institute for Brain Research, Tulsa, Oklahoma; 2 Stephenson School for Biomedical Engineering, University of Oklahoma, Norman; 3 Cardiac Arrhythmia Center, David Geffen School of Medicine at UCLA, Los Angeles, California; 4 Department of Psychiatry, University of California, San Diego; 5 Oxley College of Health Sciences, University of Tulsa, Tulsa, Oklahoma; 6 Deputy Editor, *JAMA Psychiatry*

## Abstract

**Importance:**

β-Adrenergic stimulation elicits heart palpitations and dyspnea, key features of acute anxiety and sympathetic arousal, yet no neuroimaging studies have examined how the pharmacologic modulation of interoceptive signals is associated with fear-related neurocircuitry in individuals with generalized anxiety disorder (GAD).

**Objective:**

To examine the neural circuitry underlying autonomic arousal induced via isoproterenol, a rapidly acting, peripheral β-adrenergic agonist akin to adrenaline.

**Design, Setting, and Participants:**

This crossover randomized clinical trial of 58 women with artifact-free data was conducted from January 1, 2017, to November 31, 2019, at the Laureate Institute for Brain Research in Tulsa, Oklahoma.

**Exposures:**

Functional magnetic resonance imaging was used to assess neural responses during randomized intravenous bolus infusions of isoproterenol (0.5 and 2.0 μg) and saline, each administered twice in a double-blind fashion.

**Main Outcomes and Measures:**

Blood oxygen level–dependent responses across the whole brain during isoproterenol administration in patients with GAD vs healthy comparators. Cardiac and respiratory responses, as well as interoceptive awareness and anxiety, were also measured during the infusion protocol.

**Results:**

Of the 58 female study participants, 29 had GAD (mean [SD] age, 26.9 [6.8] years) and 29 were matched healthy comparators (mean [SD] age, 24.4 [5.0] years). During the 0.5-μg dose of isoproterenol, the GAD group exhibited higher heart rate responses (*b* = 5.34; 95% CI, 2.06-8.61; *P* = .002), higher intensity ratings of cardiorespiratory sensations (*b* = 8.38; 95% CI, 2.05-14.71; *P* = .01), higher levels of self-reported anxiety (*b* = 1.04; 95% CI, 0.33-1.76; *P* = .005), and significant hypoactivation in the ventromedial prefrontal cortex (vmPFC) that was evident throughout peak response (Cohen *d* = 1.55; *P* < .001) and early recovery (Cohen *d* = 1.52; *P* < .001) periods. Correlational analysis of physiological and subjective indexes and percentage of signal change extracted during the 0.5-μg dose revealed that vmPFC hypoactivation was inversely correlated with heart rate (*r*_56_ = −0.51, adjusted *P* = .001) and retrospective intensity of both heartbeat (*r*_56_ = −0.50, adjusted *P* = .002) and breathing (*r*_56_ = −0.44, adjusted *P* = .01) sensations. Ventromedial prefrontal cortex hypoactivation correlated inversely with continuous dial ratings at a trend level (*r*_56_ = −0.38, adjusted *P* = .051), whereas anxiety (*r*_56_ = −0.28, adjusted *P* = .27) and chronotropic dose 25 (*r*_56_ = −0.14, adjusted *P* = .72) showed no such association.

**Conclusions and Relevance:**

In this crossover randomized clinical trial, women with GAD exhibited autonomic hypersensitivity during low levels of adrenergic stimulation characterized by elevated heart rate, heightened interoceptive awareness, increased anxiety, and a blunted neural response localized to the vmPFC. These findings support the notion that autonomic hyperarousal may be associated with regulatory dysfunctions in the vmPFC, which could serve as a treatment target to help patients with GAD more appropriately appraise and regulate signals of sympathetic arousal.

**Trial Registration:**

ClinicalTrials.gov Identifier: NCT02615119

## Key Points

**Question:**

Do individuals with generalized anxiety disorder (GAD) show abnormal physiological, perceptual, or neural responses during peripheral β-adrenergic stimulation that may indicate interoceptive dysfunction?

**Findings:**

In this crossover randomized clinical trial, female patients with GAD exhibited hypersensitivity to adrenergic stimulation as well as greater interoceptive sensation and diminished ventromedial prefrontal cortex activity compared with healthy participants.

**Meaning:**

This study provides evidence of dysfunctional autonomic and central nervous system contributions to the pathophysiology of GAD and suggests that the ventromedial prefrontal cortex may be a treatment target.

## Teaser

This crossover randomized clinical trial of individuals with generalized anxiety disorder examines the association between abnormal physiological, perceptual, or neural responses during peripheral β-adrenergic stimulation and interoceptive dysfunction.

## Introduction

Generalized anxiety disorder (GAD), the most common clinical manifestation of anxiety,^1^ is characterized by excessive, uncontrollable anxiety and worry persisting for at least 6 months. Patients with GAD frequently show resistance to pharmacotherapy^2^ and psychotherapy,^3^ making it one of the most difficult anxiety disorders to treat.^4,5^ Generalized anxiety disorder affects nearly twice as many women as men,^6^ and comorbidities with depression, substance use, and other anxiety disorders are common.^4,7^ Hyperarousal symptoms are often experienced by individuals with GAD,^8^ including restlessness, feeling keyed up or on edge, muscle tension, and insomnia.^9^ Patients with GAD also seek cardiologic evaluation for autonomic arousal symptoms at the same rate as those with panic disorder,^10^ but such symptoms (eg, accelerated heart rate [HR], shortness of breath, and sweating) do not consistently correlate with peripheral autonomic indexes in ambulatory studies.^11^ Consequently, their perception of physiological arousal is often mismatched with their actual physiological state,^12^ suggesting that interoceptive dysfunction is a characteristic feature of the disorder.^13^ Identifying substrates of this dysfunction that are involved in disease-modifying processes could provide novel targets for treatments that may help to overcome the high levels of anxious relapse and resistance to existing treatments.

The neural basis for anxious arousal in GAD is unclear and has not received much attention as a treatment target despite recent recommendations that modeling anxiety in humans by combining anxiety-induction procedures with neurocircuit measures provides “a key intermediate bridge between basic and clinical sciences.”^14^ Accordingly, we designed a pharmacologic protocol capable of eliciting a full range of physiological arousal responses in the cardiovascular and pulmonary systems using bolus intravenous infusions of isoproterenol, a fast-acting adrenaline analog.^15^ Isoproterenol induces transient stimulation of peripheral β_1_- and β_2_-adrenergic receptors,^16^ yielding reliable dose-dependent cardiorespiratory modulation that returns to baseline within minutes.^15^ It also has minimal blood-brain barrier penetration, making it a good probe for selectively modulating the body and measuring the afferent response inside the brain.^17,18,19^ The bolus form of administration is safe and tolerable, even in clinically anxious populations.^20,21^ Human lesion models have revealed a network of brain regions, including the insular and medial prefrontal cortices (PFCs)^22^ and the amygdala,^23^ that are critical for generating awareness of isoproterenol-induced sensations. We adapted the infusion protocol to the neuroimaging environment and demonstrated that the insular cortex is the primary region responding during isoproterenol-induced stimulation in healthy comparators (HCs) at a 2.0-μg dose.^18,24^ These functional magnetic resonance imaging (fMRI) results are corroborated by similar positron emission tomography^25^ findings.

This study used a multilevel approach to test the hypothesis that patients with GAD compared with HCs show exaggerated, dose-related subjective, physiological, and neural responses to adrenergic stimulation with isoproterenol. Support for this hypothesis would provide evidence for dysautonomia in GAD, which could be targeted in the future to develop novel interventions. To date, no functional neuroimaging studies have exposed clinically anxious individuals to isoproterenol. We hypothesized that patients with GAD would show a hypersensitivity to isoproterenol characterized by elevations in HR, interoceptive awareness of cardiorespiratory sensations, and levels of anxiety; we expected to see such differences emerge starting at a 0.5-μg dose level. On the basis of previous fMRI studies,^18,24^ we anticipated that this physiological and subjective hypersensitivity in GAD would be reflected by heightened neural responses in the insular cortex.

## Methods

### Participants

Diagnostic grouping of participants in this crossover randomized clinical trial was based on *DSM-IV* or *DSM-5* criteria using the Mini-International Neuropsychiatric Interview.^26^ Additional inclusion criteria required patients with GAD to have scores greater than 7 and 10, respectively, on the Overall Anxiety Severity and Impairment Scale^27^ and GAD-7^28^ scale, indicative of clinically significant anxiety. Current or prior cardiorespiratory illness, including asthma, or other major psychiatric disorders (eMethods 1 in Supplement 1) were exclusionary. Although comorbid depression and anxiety disorders were allowed, panic disorder was exclusionary to reduce potential dropout associated with isoproterenol-induced panic anxiety.^29^ Selected psychotropic agents were allowed provided there was no change in dosage 4 weeks before the MRI. Demographic matching variables included self-reported sex, age, and measured body mass index (BMI) (calculated as weight in kilograms divided by height in meters squared). Self-reported race and ethnicity were recorded as required by the funding agency. All study procedures were approved by the Western Institutional Review Board, and all participants provided written informed consent before participation. This study followed the Consolidated Standards of Reporting Trials (CONSORT) reporting guideline (study protocol is given in Supplement 2), and data were collected from January 1, 2017, to November 31, 2019, at the Laureate Institute for Brain Research, Tulsa, Oklahoma.

### Experimental Protocols

We measured parametric physiological and blood oxygen level–dependent (BOLD) changes during randomized, double-blind intravenous bolus infusions of isoproterenol hydrochloride (0.5 and 2 μg) or saline administered 60 seconds into each MRI infusion scan (eFigure 1 in Supplement 1). Each infusion condition was repeated once for a total of 6 infusion scans administered via a randomized crossover procedure (eMethods 2 in Supplement 1). Cardiac and respiration waveforms were measured simultaneously with fMRI using, respectively, a pulse oximeter attached to a nondominant finger and a thoracic respiration belt, both sampled at 40 Hz. Participants continuously rated changes in perceived cardiorespiratory intensity by rotating an MRI-compatible dial (Current Designs Inc) with their dominant hand throughout each 240-second scan using a visual analog scale ranging from 0 (none or normal) to 10 (most ever) (eFigure 2 in Supplement 1). After each infusion, participants retrospectively reported their perceived cardiac and respiratory sensation intensity as well as anxiety and excitement using an 11-point scale.^24^ Before the MRI session, we administered 2 single-blind infusions (saline and 1.0 μg of isoproterenol) to familiarize participants with the infusion procedure (eMethods 2 in Supplement 1).

### Behavioral and Physiological Analysis

We indirectly estimated β-adrenergic receptor sensitivity for each participant by calculating the chronotropic dose 25 (CD25), a standard parameter that reflects the dose required to increase HR by 25/min^16^ according to the formula provided by Mills et al.^30^ We tested for group differences in CD25 via 2-tailed, independent *t* test. Interoceptive detection rates were assessed via χ^2^ test (eMethods 3 and eFigure 4 in Supplement 1). We used linear mixed-effects regression to examine epoch effects of group and dose on HR, respiratory volume variability, respiratory results (reported in eResults 1, eFigure 3, and eTable 5 in Supplement 1), interoceptive awareness (continuous dial ratings, retrospective heartbeat intensity, and respiratory intensity ratings), and retrospective anxiety and excitement ratings. Fixed effects for group (HCs and patients with GAD) and dose were included for post-MRI ratings and cross-correlations between HR and dial ratings (eMethods 3, eResults 2, eFigure 6, and eTables 6 and 7 in Supplement 1) as well as their second-level interactions. All linear mixed-effects models included fixed effects for participant age, BMI, and their group variable interaction (eMethods 3 in Supplement 1).

### MRI Data Acquisition

We acquired anatomical, T1-weighted, magnetization-prepared, rapid gradient-echo sequence images and T2*-weighted BOLD contrast images via an echoplanar sequence in a 3-T scanner with an 8-channel head coil (GE MR750; GE Healthcare) (eMethods 4 in Supplement 1).

### fMRI Analysis

Data preprocessing and analyses were performed in AFNI.^31^ Preprocessing steps included despiking, slice time correction, spatial smoothing (6-mm full width at half maximum gaussian kernel), coregistation to the first volume, and normalization to Talairach space (eMethods 5 in Supplement 1). Temporal fluctuations of cardiac and respiratory frequencies and their first-order harmonics were removed from the BOLD signal using RETROICOR^32^ implemented via custom code in MATLAB (MathWorks).^24^ Individual-level percentage of signal change (PSC) maps of BOLD response to cardiorespiratory stimulation were generated from residual images by contrasting epoch-averaged signals for isoproterenol against the baseline period (first 45 seconds) preceding each infusion. On the basis of previous studies,^18,24^ we defined 4 discrete epochs of interest as follows (eFigure 1 in Supplement 1): the anticipatory period (20 seconds) after infusion delivery but preceding the onset of isoproterenol effects, the peak effect period (40 seconds), and early (60 seconds) and late (62 seconds) recovery periods. We performed whole-brain voxelwise *t* tests contrasting group-averaged PSC maps for each epoch to examine potential differences between the GAD and HC groups using *3dttest*++ in AFNI (eMethods 6 in Supplement 1). In addition, to better understand how the temporal dynamics of isoproterenol’s effects on BOLD response, HR, and sensation rating trajectories might differ between the GAD and HC groups without predefining epochs, we performed a multivariate, sparse functional principal components analysis. Methodologic details for this technique can be found in eMethods 7 and eFigure 7, and the results are found in eResults 4, eFigure 8, and eTables 8-12 in Supplement 1.

### Multilevel Correlational Analysis and Linear Modeling of Outcomes

To assess dimensional associations between outcomes, we conducted post hoc, multiple comparison–corrected Pearson correlations across diagnostic groups for cardiorespiratory, subjective (continuous or retrospective), and neural responses (eMethods 3 in Supplement 1). To evaluate whether diagnostic group affects the associations between outcome variables, we tested for differences in slopes estimated by linear models. Specifically, we generated linear models for BOLD PSC activity and HR or subjective dial responses in the form of lm(PSC − HR × group) and evaluated the interaction term.

## Results

**Figure 1.**
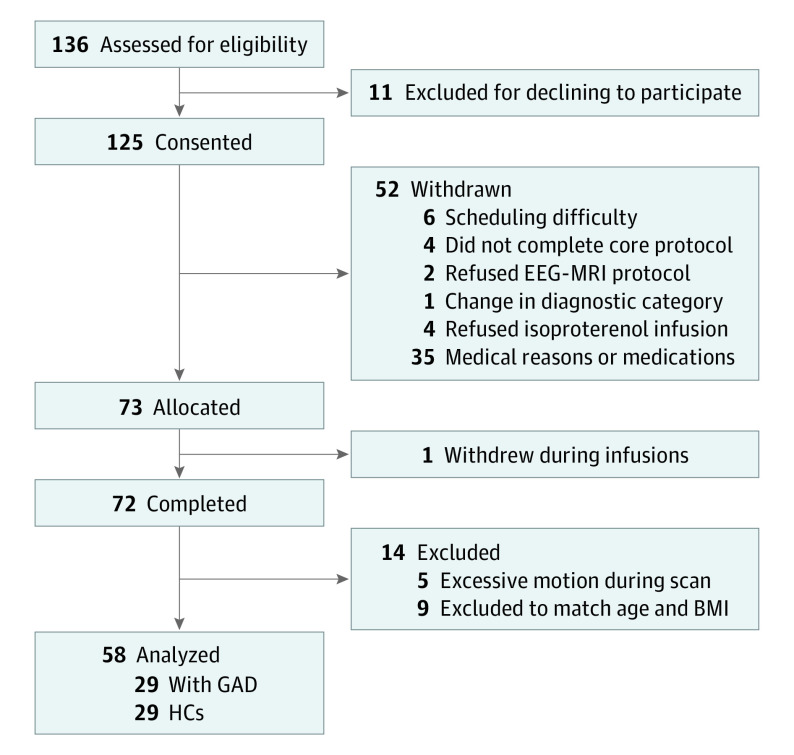
CONSORT Diagram BMI indicates body mass index; EEG, electroencephalography; GAD, generalized anxiety disorder; HC, healthy comparator; and MRI, magnetic resonance imaging.

**Figure 2.**
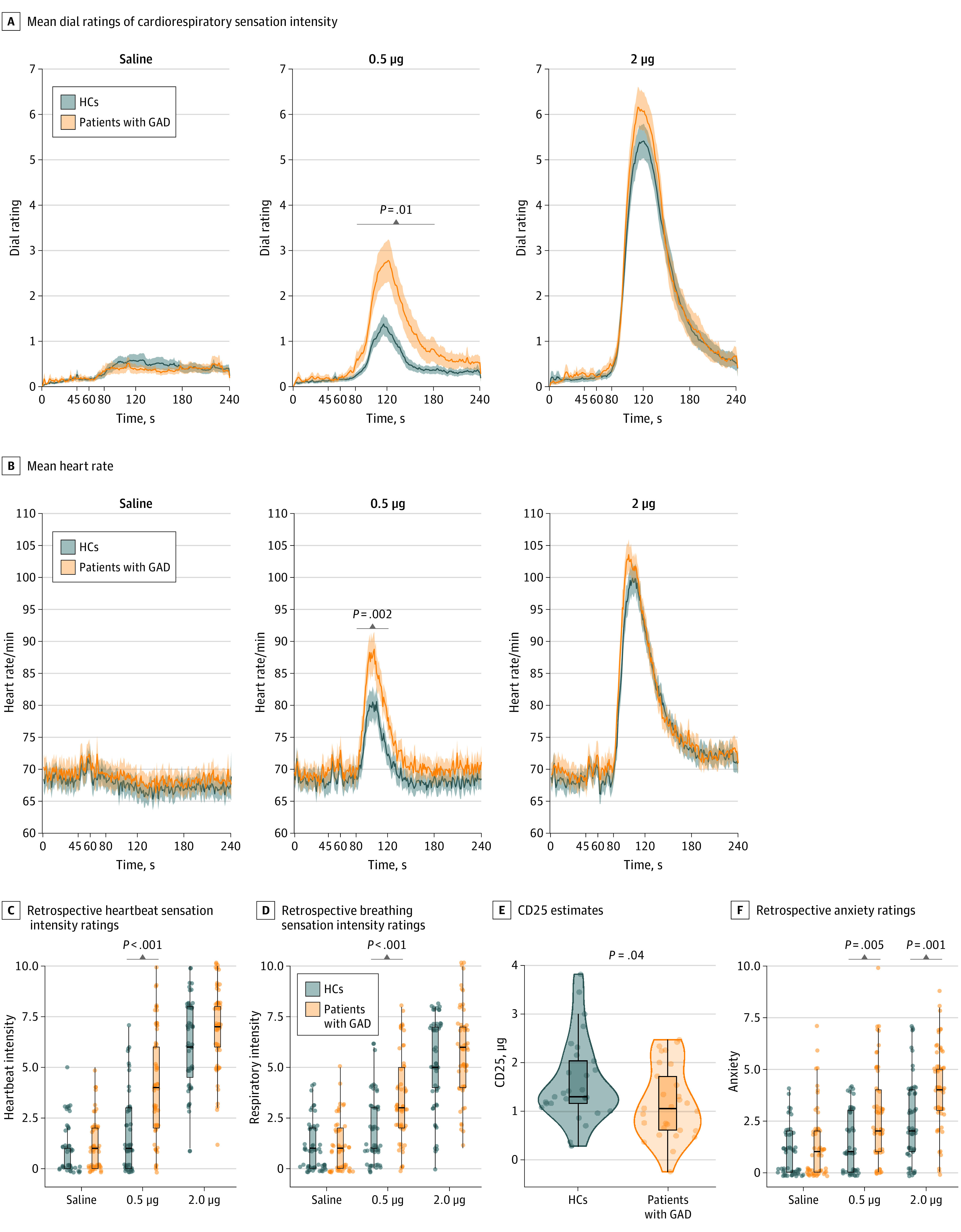
Physiological, Perceptual, and Affective Responses During Isoproterenol and Saline Infusions Box plots are shown representing median (thick line) and 25th and 75th quartiles (thin lines) with whiskers extending to 1.5 × the IQR. Chronotropic dose 25 (CD25) values reflect the dose required to elevate the heart rate by 25/min. GAD indicates general anxiety disorder; HCs, healthy comparators.

Of the 58 female study participants, 29 had GAD (mean [SD] age, 26.9 [6.8] years) and 29 were matched HCs (mean [SD] age, 24.4 [5.0] years) based on self-reported age and measured BMI (Table and Figure 1; eTable 1 in Supplement 1). The GAD group reported greater retrospective intensity of both heartbeat (estimate [SE] *b* = 2.21 [0.44]; 95% CI, 1.35-3.07; *t*_278.81_ = 5.04; *P* < .001) and breathing sensations (estimate [SE] *b* = 1.51 [0.41]; 95% CI, 0.72-2.32; *t*_276_ = 3.70; *P* < .001) during 0.5-μg infusions (Figure 2C and D). Anxiety ratings for those with GAD were also significantly greater than HCs at both the 0.5-μg dose (estimate [SE] *b* = 1.04 [0.37]; 95% CI, 0.33-1.76; *t*_281.32_ = 2.84; *P* = .005) and 2.0-μg dose (estimate [SE] *b* = 1.22 [0.36]; 95% CI, 0.51-1.94; *t*_281.11_ = 3.36; *P* =  001) doses (Figure 2F), whereas no main or interaction effects were seen for excitement at either dose (eTable 4 in Supplement 1).

**Table.** Descriptive and Inferential Statistics for Demographic and Diagnostic Variables

Variable	Mean (SD)		*t* Value (*df*)	*P* value
	Patients with GAD (n = 29)	HCs (n = 29)		
Age, y	26.9 (6.8)	24.4 (5.0)	1.61 (51.62)	.11
BMI	25.64 (4.59)	24.04 (3.12)	1.55 (49.33)	.13
GAD-7	13.52 (3.41)	1.03 (1.52)	−18.01 (38.76)	<.001
OASIS	10.79 (2.23)	1.17 (1.56)	−19.06 (50.16)	<.001
ASI-3	27.62 (14.46)	7.55 (4.26)	−7.17 (32.82)	<.001
PHQ-9	11.21 (4.95)	0.72 (1.10)	−11.13 (30.75)	<.001

Abbreviations: ASI-3, Anxiety Sensitivity Index 3; BMI, body mass index (calculated as weight in kilograms divided by height in meters squared); GAD, Generalized Anxiety Disorder; GAD-7, 7-item Generalized Anxiety Disorder scale; HCs, healthy comparators, OASIS, Overall Anxiety Severity and Impairment Scale; PHQ-9, 9-item Patient Health Questionnaire.

The GAD group showed greater adrenergic sensitivity to isoproterenol than HCs as indicated by smaller CD25 values (GAD group = 1.17 μg; HC group = 1.61 μg; Cohen *d* = 0.55; 95% CI, 0.02-0.86; *t*_54.97_ = 2.10; *P* = .04) (Figure 2E). Epoch-based linear mixed-effects models for HR suggested that this effect of GAD was driven primarily by sensitivity at the peak of the 0.5-μg dose (estimate [SE] *b* = 5.34 [1.68]; 95% CI, 2.06-8.61; *t*_1650.04_ = 3.17; *P* = .002; mean GAD HR increase of 13 beats per minute, mean HC HR increase of 7 beats per minute) (Figure 2B; eTable 2 in Supplement 1), which corresponded with greater perception of cardiorespiratory intensity during the peak (estimate [SE] *b* = 8.38 [3.26]; 95% CI, 2.05-14.71; *t*_1650.55_ = 2.57; *P* = .01) and early recovery (estimate [SE] *b* = 9.11 [3.26]; 95% CI, 2.77-15.45; *t*_1650.04_ = 2.80; *P* = .005) periods (Figure 2A; eTable 3 in Supplement 1).

**Figure 3.**
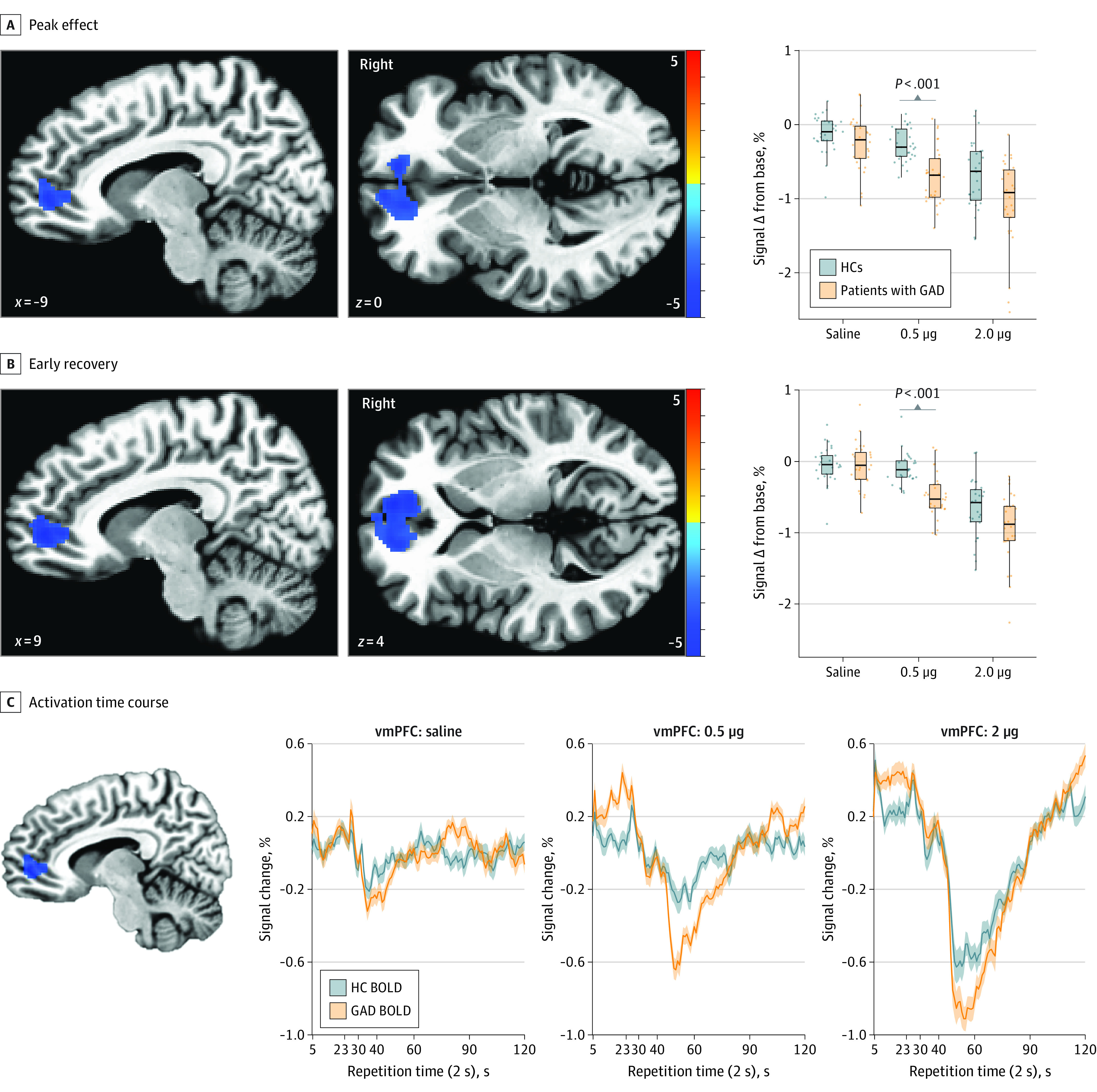
Group Differences in Ventromedial Prefrontal Cortex Response From Whole-Brain Analyses A, Significant group activation differences during the peak response period were only found during administration of the 0.5-μg dose at a voxelwise threshold of *P* < .001 and a 95% false-positive rate cluster correction. B, Significant group activation differences during the early recovery period were only found during administration of the 0.5-μg dose. Right insets show the percentage of signal change above baseline for each dose. C, Mean activitation time course for the significant ventromedial prefrontal cortex (vmPFC) region (blue cluster from peak period) identified during the 0.5-μg infusion. Shaded lines indicate SEMs. BOLD indicates blood oxygenation level–dependent response; GAD, generalized anxiety disorder; and HC, healthy comparator.

**Figure 4.**
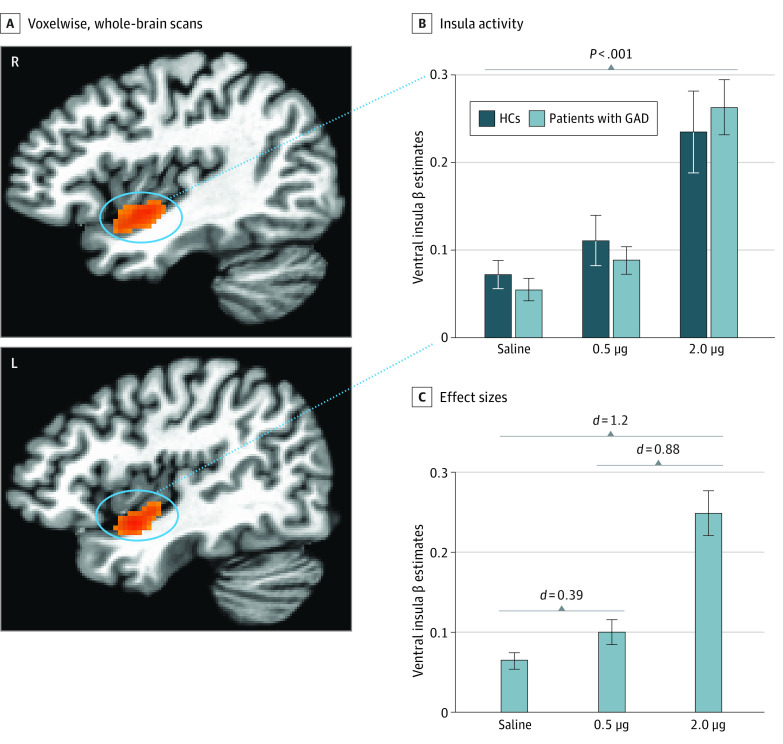
Insular Cortex Responses During Isoproterenol and Saline Infusion A, Increased activity localized within bilateral clusters of ventral insular cortex was observed across all participants via voxelwise, whole-brain analysis during the peak effect epoch of the 2.0-μg isoproterenol infusion. B, No significant differences were found between groups within the ventral insular cortex clusters at each dose. The 2.0-μg infusion elicited a significantly larger insula response than the saline and the 0.5-μg infusion. The clusters identified at 2.0 μg were used as masks for extracting signal change for each dose and the saline infusions based on prior studies^18,24^ in which the 2.0-μg dose proved to be sensitive at eliciting insula activity in healthy individuals. C, Cohen *d* effect sizes when comparing each isoproterenol dose with saline. Error bars indicate SEMs. GAD indicates generalized anxiety disorder; HC, healthy comparator.

Whole-brain results are reported at the *P* < .001 level with a 5% false-positive rate cluster correction. In alignment with the physiological observations of dose-specific hypersensitivity, we observed significant group differences during the peak and early recovery epochs during the 0.5-μg but not 2.0-μg infusions (Figure 3A and B). Specifically, the GAD group showed significantly decreased BOLD signal changes in 2 clusters during the peak response period: the bilateral ventromedial prefrontal cortex (vmPFC) extending to the rostral anterior cingulate cortex (ACC) (k = 601, center of mass: x = −6.9, y = 44.6, z = 0.1; Cohen *d* = 1.55; *P* < .001) and the left angular gyrus extending into the precuneus (k = 246, center of mass: x = −37, y = −65.3, z = 36.2) (eFigure 5 in Supplement 1). During the early recovery period, the vmPFC again showed greater attenuation for the GAD group (k = 779, center of mass: x = −2.2, y = 45.2, z = 1.4; Cohen *d* = 1.52; *P* < .001). The whole-brain analysis revealed no other significant regional group differences during any other epoch but identified effects of dose for the insular cortex across all participants. This dose-dependent activation was found bilaterally in the ventral mid insula during the peak and early recovery epochs relative to the preinfusion baseline at both 0.5 and 2.0 μg (Figure 4). Potential effects of medication status on the linear mixed-effects models were assessed by generating models for the whole-brain, physiological, and subjective responses after removing the 6 medicated patients of the GAD group. For each response modality, similar group differences were observed at the 0.5-μg dose in the unmedicated GAD subgroup (eResults 3 in Supplement 1).

Correlational analysis of physiological and subjective indexes and PSC extracted during the 0.5-μg dose revealed that vmPFC hypoactivation was inversely correlated with HR (*r*_56_ = −0.51, adjusted *P* = .001) and retrospective intensity of both heartbeat (*r*_56_ = −0.5, adjusted *P* = .002) and breathing (*r*_56_ = −0.44, adjusted *P* = .01) sensations. The vmPFC hypoactivation correlated inversely with dial ratings at a trend level (*r*_56_ = −0.38, adjusted *P* = .051), whereas anxiety (*r*_56_ = −0.28, adjusted *P* = .27) or CD25 (*r*_56_ = −0.14, adjusted *P* = .72) showed no such association (eFigure 9 and eTable 13 in Supplement 1). Evaluation of group differences in slopes estimated by linear models of the associations between voxelwise vmPFC PSC and either HR (estimate [SE] *b* = −0.02 [0.01]; *t*_54_ = −1.73; *P* = .09) or subjective dial response (estimate [SE] *b* = −0.01 [0.01]; *t*_54_ = −0.66; *P* = .51) indicated that the associations between these outcomes were not affected by group.

## Discussion

We used peripheral β-adrenergic stimulation with isoproterenol to evaluate physiological, subjective, and neural responses during fMRI in women with GAD compared with HCs. As expected, this manipulation evoked dose-dependent increases in cardiorespiratory parameters across all participants. However, the GAD group exhibited hypersensitivity to the lower dose of isoproterenol across all response modalities. Specifically, during the peak window of the 0.5-μg dose, the GAD group demonstrated significant elevations in HR while also reporting significantly more intense cardiorespiratory sensations during this dose and significantly more anxiety than HCs during both doses. Whole-brain fMRI analyses revealed significant group differences only during the 0.5-μg infusion, with the GAD group exhibiting bilateral vmPFC hypoactivation throughout the peak and early recovery epochs and left inferior parietal cortex hypoactivation during the peak epoch compared with HCs. Of note, vmPFC activation differences were moderately to strongly correlated with HR and cardiorespiratory self-report during the 0.5-μg infusion. The lack of group differences in physiological or neural responses to saline or the 2.0-μg infusion highlighted the GAD group’s sensitivity to sympathetic arousal signals during lower levels of adrenergic stimulation.

The present experimental medicine findings provide novel evidence of autonomic and central nervous system contributions to the interoceptive pathophysiological mechanisms of GAD. Although sympathetic hyperarousal has not been prominently associated with GAD in ambulatory or laboratory studies,^11,33,34^ such studies did not rely on parametric receptor stimulation. Lower CD25 values in the GAD group suggest a greater peripheral β-adrenergic receptor density, which could explain their exaggerated physiological response to adrenergic stimulation (ie, a left-shifted drug potency effect). At this dose, we also saw altered brain responses in the vmPFC, a key node of the central autonomic network^35,36^ with multisynaptic connections to viscerosensory and visceromotor autonomic ganglia.^37^ The blunted vmPFC activation together with the exaggerated self-reported and physiological responses support the idea that a lack of top-down regulation in the presence of a bottom-up sympathetic nervous system stimulation elicits an internal state that further promotes an anxious response. Cortical thinning of this area has been seen in patients with GAD compared with HCs and was positively correlated with worry and trait anxiety across groups.^38^ The vmPFC and ACC are highly connected with the dorsolateral PFC and have been observed to be inhibited by worry induction.^39^ This circuit could help to explain the chronic high-anxiety state typical in GAD.

Because no group differences were found in vmPFC hypoactivation at high doses, the effect at low doses may also represent a dysfunctional central regulatory threshold^40,41,42^ in GAD. These neural effects cannot be entirely attributable to a greater propensity of patients with GAD to focus their attention on cardiorespiratory sensations because no significant between-group differences were found in brain activation during the saline condition. Despite the causal nature of our peripheral afferent manipulation, pinpointing the propagation and controllers of homeostatic signals through the afferent and efferent limbs of the brain-body (ie, cybernetic^43,44^) feedback loop remains challenging. Computational models of homeostatic and allostatic interoceptive regulation^45^ reflect one approach for differentiating sensory inputs from selected actions via incorporation of hierarchical precision-weighted predictions and prediction errors.^46^ Thus, whether the GAD group’s more vigorous reaction to lower levels of stimulation stems from abnormal peripheral inputs (ie, overabundance of cardiovascular β-adrenergic receptors), aberrant visceromotor responses (ie, a dysfunctional central controller), or interacting peripheral and central processes awaits determination.

To our knowledge, this is the first neuroimaging study to examine how the direct modulation of interoceptive signals influences fear-related (ie, vmPFC) neurocircuitry in individuals with clinical anxiety. Converging lesion^47,48,49^ and functional neuroimaging^50,51,52^ evidence implicates the vmPFC in the regulation of negative affect and worry, including the suppression and spontaneous recovery of fear.^53,54,55^ Further work has suggested that the vmPFC provides a signal^56^ that previously threatening stimuli may be reconsidered as safe.^57^ Indeed, the portion of the vmPFC we found to have the most disruption during low levels of adrenergic stimulation overlaps with the region most correlated with safety.^58^ In addition, vmPFC hypoactivity is related to difficulty discerning safety from threat,^59^ deficient top-down control in individuals with anxiety during threat-related distractors,^60^ abnormal emotion processing across psychiatric disorders,^61^ and worry severity in GAD.^52^ Of note, “categorizing and assigning value to interoceptive sensations through the filter of one’s autobiographical narrative”^62^ is a key attributional process theorized to be mediated by the vmPFC. Our results may thus reflect a deficient self-appraisal capacity in GAD to adaptively assign meaning during interoceptively driven anxious arousal. This argument is supported by the vmPFC’s role in regulating the physiological state of the body based on self-relevant contexts.^63,64^ Potential clinical applications from this work include pharmacologic or neuromodulation targeting of vmPFC responses (eg, via real-time fMRI neurofeedback or transcranial focused ultrasonography) to determine their effect on anxious rumination in GAD.

The insula was activated in both groups during isoproterenol infusions, consistent with an insular mapping of cardiorespiratory states,^24^ although, contrary to our expectations, we did not observe significant group differences. Given the exaggerated perceptual and physiological responses observed in GAD, perhaps dysfunction within the vmPFC led to failures to appropriately appraise sympathetic arousal signals from the insula, which could have contributed to their heightened reports of anxiety. This explanation seems plausible given that the vmPFC assigns valence to different states of arousal,^62,65^ particularly through its reciprocal anatomical connections with the insula,^66^ the primary cortical recipient of afferent viscerosensory signals.^67,68,69^ Indeed, previous studies^70,71^ have shown reduced functional connectivity between both regions in individuals with anxiety under resting conditions. Thus, dysregulation within vmPFC-to-insula neurocircuitry may entail a failure to constrain sympathetic arousal signals, resulting in downstream consequences, such as increased worry, rumination, and anxiety^72^; such a process has been observed during periods of sympathetic arousal associated with anxious rumination.^73,74^

### Limitations

This study has limitations, including the limited number of participants, a female-only sample, and psychotropic medication allowance (eDiscussion in Supplement 1).

## Conclusions

In this crossover randomized clinical trial, women with GAD showed autonomic hypersensitivity during low levels of adrenergic stimulation characterized by an elevated HR, heightened interoceptive awareness, increased anxiety, and a blunted neural response localized to the vmPFC. Autonomic hyperarousal may be linked to regulatory dysfunctions in the vmPFC, which could serve as a treatment target to help patients with anxiety more appropriately appraise and regulate sympathetic arousal signals emanating from the autonomic nervous system.

## Notes

Supplement 1. **eMethods 1.** Participants **eMethods 2.** Experimental Protocol **eMethods 3.** Primary and Exploratory Behavioral and Physiological Data Analysis **eMethods 4.** MRI Data Acquisition **eMethods 5.** fMRI Preprocessing **eMethods 6.** Whole-Brain fMRI Group Analysis **eMethods 7.** Longitudinal Time Series Analysis With Regions of Interest **eFigure 1.** Infusion Epochs Used in the Isoproterenol fMRI Analysis **eFigure 2.** Subjective Response Assessment **eFigure 3.** Respiratory Volume Variability During Isoproterenol Infusion **eFigure 4.** Interoceptive Detection Rates **eFigure 5.** Group Difference in Left Parietal Cortex Activity **eFigure 6**. Maximum Cross-correlations Between Heart Rate and Dial Response by Dose in Unmedicated GAD Relative to HC **eFigure 7.** Overlap Between Whole-Brain vmPFC Cluster and Meta-analysis–Derived ROIs **eFigure 8.** Multivariate Sparse Functional Principal Component Analysis (mSFPCA) Model Fits to Raw Data by Dose and Group **eFigure 9.** vmPFC PSC Correlations With Cardiorespiratory and Subjective Variables During the Peak Effect at 0.5μg Isoproterenol **eResults 1.** Respiratory Volume Variability (RVV) **eResults 2.** Interoceptive Awareness: Detection and Accuracy Measures **eResults 3**. Reanalysis of Primary Outcomes With Medicated Participants Removed **eResults 4**. Longitudinal Time-Series Analysis **eTable 1.** Diagnostic Comorbidities and Psychotropic Medication Status of Generalized Anxiety Disorder and Healthy Comparator Participants **eTable 2.** LME for Mean Heart Rate by Group and Dose for Selected Isoproterenol Time-Course Epochs **eTable 3.** LME for Continuous Cardiorespiratory Dial Ratings by Group and Dose for Selected Isoproterenol Time-Course Epochs **eTable 4.** LME for Retrospective Ratings by Group and Dose **eTable 5.** LME for Respiratory Volume Variability by Group and Dose for Selected Isoproterenol Time-Course Epochs **eTable 6.** LME for Cross Correlations (CC) Between Real-Time Dial Responses and Heart Rate **eTable 7.** LME for Cross Correlations (CC) Between Continuous Dial Responses and Respiratory Volume Variability **eTable 8.** LMEs for FPC Score Reflecting Signal Change Timeseries for Meta-analysis–Derived vmPFC ROIs **eTable 9.** LME for Mean BOLD PSC for the Meta-analysis–Derived Autonomic vmPFC ROI **eTable 10.** LME for BOLD PSC for the Meta-analysis–Derived Self-processing vmPFC ROI **eTable 11.** LME for BOLD PSC for the Meta-analysis–Derived Valence vmPFC ROI **eTable 12.** LMEs for Heart Rate and Dial Response FPC Scores **eTable 13.** Multilevel Correlations Between vmPFC Activity, Cardiac, and Subjective Responses During 0.5μg Isoproterenol Dose **eDiscussion.** Study Limitations
eReferences

Supplement 2.
Trial Protocol
