# Supplementary material for: Association of Generalized Anxiety Disorder With Autonomic Hypersensitivity and Blunted Ventromedial Prefrontal Cortex Activity During Peripheral Adrenergic Stimulation: A Randomized Clinical Trial
Source: JAMA Psychiatry. 2022 Feb 2;79(4):323–32. doi: 10.1001/jamapsychiatry.2021.4225 (PMC8811711; doi:10.1001/jamapsychiatry.2021.4225)
Supplement: Supplement 1. — eMethods 1. Participants eMethods 2. Experimental Protocol eMethods 3. Primary and Exploratory Behavioral and Physiological Data Analysis eMethods 4. MRI Data Acquisition eMethods 5. fMRI Preprocessing eMethods 6. Whole-Brain fMRI Group Analysis eMethods 7. Longitudinal Time Series Analysis With Regions of Interest eFigure 1. Infusion Epochs Used in the Isoproterenol fMRI Analysis eFigure 2. Subjective Response Assessment eFigure 3. Respiratory Volume Variability During Isoproterenol Infusion eFigure 4. Interoceptive Detection Rates eFigure 5. Group Difference in Left Parietal Cortex Activity eFigure 6. Maximum Cross-correlations Between Heart Rate and Dial Response by Dose in Unmedicated GAD Relative to HC eFigure 7. Overlap Between Whole-Brain vmPFC Cluster and Meta-analysis–Derived ROIs eFigure 8. Multivariate Sparse Functional Principal Component Analysis (mSFPCA) Model Fits to Raw Data by Dose and Group eFigure 9. vmPFC PSC Correlations With Cardiorespiratory and Subjective Variables During the Peak Effect at 0.5μg Isoproterenol eResults 1. Respiratory Volume Variability (RVV) eResults 2. Interoceptive Awareness: Detection and Accuracy Measures eResults 3. Reanalysis of Primary Outcomes With Medicated Participants Removed eResults 4. Longitudinal Time-Series Analysis eTable 1. Diagnostic Comorbidities and Psychotropic Medication Status of Generalized Anxiety Disorder and Healthy Comparator Participants eTable 2. LME for Mean Heart Rate by Group and Dose for Selected Isoproterenol Time-Course Epochs eTable 3. LME for Continuous Cardiorespiratory Dial Ratings by Group and Dose for Selected Isoproterenol Time-Course Epochs eTable 4. LME for Retrospective Ratings by Group and Dose eTable 5. LME for Respiratory Volume Variability by Group and Dose for Selected Isoproterenol Time-Course Epochs eTable 6. LME for Cross Correlations (CC) Between Real-Time Dial Responses and Heart Rate eTable 7. LME for Cross Correlations (CC) Between Continuous Dial Responses and Respir [file jamapsychiatry-e214225-s001.pdf]

Supplementary Online Content

Teed AR, Feinstein JS, Puhl M, et al. Association of generalized anxiety disorder with autonomic hypersensitivity and blunted ventromedial prefrontal cortex activity during peripheral adrenergic stimulation: a randomized clinical trial. *JAMA Psychiatry.* Published online February 2, 2022. doi:10.1001/jamapsychiatry.2021.4225

eMethods 1. Participants
eMethods 2. Experimental Protocol
eMethods 3. Primary and Exploratory Behavioral and Physiological Data Analysis
eMethods 4. MRI Data Acquisition
eMethods 5. fMRI Preprocessing
eMethods 6. Whole-Brain fMRI Group Analysis
eMethods 7. Longitudinal Time Series Analysis With Regions of Interest
eFigure 1. Infusion Epochs Used in the Isoproterenol fMRI Analysis
eFigure 2. Subjective Response Assessment
eFigure 3. Respiratory Volume Variability During Isoproterenol Infusion
eFigure 4. Interoceptive Detection Rates
eFigure 5. Group Difference in Left Parietal Cortex Activity
eFigure 6. Maximum Cross-correlations Between Heart Rate and Dial Response by Dose in Unmedicated GAD Relative to HC
eFigure 7. Overlap Between Whole-Brain vmPFC Cluster and Meta-analysis–Derived ROIs
eFigure 8. Multivariate Sparse Functional Principal Component Analysis (mSFPCA) Model Fits to Raw Data by Dose and Group
eFigure 9. vmPFC PSC Correlations With Cardiorespiratory and Subjective Variables During the Peak Effect at 0.5µg Isoproterenol
eResults 1. Respiratory Volume Variability (RVV)
eResults 2. Interoceptive Awareness: Detection and Accuracy Measures
eResults 3. Reanalysis of Primary Outcomes With Medicated Participants Removed
eResults 4. Longitudinal Time-Series Analysis
eTable 1. Diagnostic Comorbidities and Psychotropic Medication Status of Generalized Anxiety Disorder and Healthy Comparator Participants
eTable 2. LME for Heart Rate by Group and Dose for Selected Isoproterenol Time-Course Epochs
eTable 3. LME for Continuous Cardiorespiratory Dial Ratings by Group and Dose for Selected Isoproterenol Time-Course Epochs
eTable 4. LME for Retrospective Ratings by Group and Dose
eTable 5. LME for Respiratory Volume Variability by Group and Dose for Selected Isoproterenol Time-Course Epochs
eTable 6. LME for Cross Correlations (CC) Between Real-Time Dial Responses and Heart Rate
eTable 7. LME for Cross Correlations (CC) Between Continuous Dial Responses and Respiratory Volume Variability
eTable 8. LMEs for FPC Score Reflecting Signal Change Timeseries for Meta-analysis–Derived vmPFC ROIs
eTable 9. LME for BOLD PSC for the Meta-analysis–Derived Autonomic vmPFC ROI
eTable 10. LME for BOLD PSC for the Meta-analysis–Derived Self-processing vmPFC ROI
eTable 11. LME for BOLD PSC for the Meta-analysis–Derived Valence vmPFC ROI
eTable 12. LMEs for Heart Rate and Dial Response FPC Scores
eTable 13. Multilevel Correlations Between vmPFC Activity, Cardiac, and Subjective Responses During 0.5µg Isoproterenol Dose
eDiscussion 1. Study Limitations
eReferences

This supplementary material has been provided by the authors to give readers additional information about their work.

© 2022 Teed AR et al. *JAMA Psychiatry*.

eMethods 1. Participants

One hundred thirty-six participants were assessed for eligibility, following study recruitment from outpatients receiving services at the Laureate Psychiatric Clinic and Hospital as well as via newspaper, radio, flyer, and social media advertisements in the Tulsa metropolitan area. Those with current/prior schizophrenia, bipolar disorder, or obsessive-compulsive disorder were excluded. Participants were allowed to continue use of selected psychotropic medications but were instructed not to take any analgesic or other over the counter (OTC) medication within 48 hours of the experimental session and no benzodiazepines, beta-blockers, or tetrahydrocannabinol (THC) containing products within 24 hours of scanning. No participants tested positively for THC or benzodiazepines on the day of scanning. They were further instructed not to have nicotine on the day of the experimental session and to limit their caffeine consumption (i.e., avoid caffeine it was not a daily routine for the participant, and if it was, to reduce their usual amount by half).

Eleven candidates declined to participate and the remaining 125 provided informed written consent. Seventeen participants withdrew prior to the intervention for various reasons (scheduling difficulty, incomplete baseline assessment, or study refusal). Thirty-five additional participants were excluded due to contraindicated medical conditions.

Medical contraindications included: 1) (a) cardiac disease (e.g. bradycardia <40 bpm, any history of diagnoses EKG abnormalities or evidence of 12-lead EKG abnormalities during baseline visit or evidence of frequent premature ventricular contractions during baseline visit), (b) respiratory disease (asthma, pneumonia), (c) endocrine disease (hypothyroidism, diabetes), (d) neurological disease (seizure disorder or reporting a seizure within the last year, head injury with sustained loss of consciousness and memory loss), (e) pain disorder (f) history of hepatic or renal failure, (g) gastrointestinal, hematologic, rheumatologic, or metabolic disease, 4) pregnancy as verified by urine test, 5) intrauterine device (IUD) posing a scanning risk, 6) ages 18 to 55 years, 7) excessive BMI (BMI > 35) preventing scanner entry, 8) MRI contraindications including: (a) cardiac pacemaker, (b) metal fragments in eyes/skin/body (shrapnel), (c) aortic/aneurysm clips, (d) prosthesis, (e) by-pass surgery/coronary artery clips, (f) heart valve replacement, (g) shunt (ventricular or spinal), (h) implanted electrodes, (i) MRI contraindicated metal plates/pins/screws/ wires, or neuro/bio-stimulators (TENS unit), (j) persons with a history of professional metal working/welding who showed evidence of intraocular metal on orbital x-ray, history of eye surgery/eyes washed out because of metal, (k) inability to lie still on one's back for a 1-2 hours, (l) prior neurosurgery, (m) tattoos or cosmetic makeup with metal dyes, (n) unwillingness or inability to remove body piercings, 9) medications that affect the hemodynamic response (e.g. acetazolamide, excessive caffeine intake > 1000 mg per day, 10), non-correctable vision or hearing problems, or 11) vital sign abnormalities during the screening visit (e.g. systolic blood pressure >160 mmHg, diastolic blood pressure >100 mmHg, resting pulse >100 beats per minute).

Of the 73 allocated to the intervention, 72 completed it. One GAD participant withdrew during the infusions due to panic anxiety. Five participants were excluded due to excessive head motion resulting in low data quality (four HCs and one GAD), eight HCs were excluded during the matching process based on age and BMI, and one GAD was excluded for having a BMI too high to appropriately match with a healthy comparator. All participants were financially compensated for their participation.

eMethods 2. Experimental Protocol

Isoproterenol was obtained from Valeant Pharmaceuticals, Laval, QC, Canada and infusions were prepared into 3 mL boluses by a pharmacist who was unblinded. The isoproterenol infusion administration procedure mirrored our previous fMRI cross-over protocol in healthy volunteers[1], with infusions administered by a nurse seated beside the scanning bed inside of the MRI room who was blinded to infusion condition. For safety monitoring purposes, cardiac rhythm was also recorded continuously using two MRI-compatible ECG leads (lead I and II configuration) (GE Healthcare, Waukesha, WI, USA). These rhythms and vital signs were monitored continuously by the nurse delivering the infusions. As an additional safety precaution, condition order was available to the research assistant sitting in the control room, who was blinded to the study hypotheses.

The randomized sequences for dose order were individually predetermined via a random number generator prior to beginning study sample recruitment. Upon recruitment of each participant, a 3rd party registered nurse (i.e., uninvolved in infusion administration or data collection) selected a randomization code from a set of randomizations generated by the senior study author. The 3rd party nurse then arranged the bolus doses in the predetermined order, and after obtaining verification of this order by a second 3rd party nurse (or nursing assistant), covered the labels. Selection of a randomization order for a given participant was not determined by group membership and occurred in sequential order from the list of randomization orders.

On arrival participants ate a 300 Calorie snack. Each participant was led through a training session approximately one hour before fMRI scanning. During this session, the participant was instructed that they would receive both isoproterenol and saline infusions at different points during the scan. Participants were informed that "you may notice an increase in your heartbeat sensations, and/or may notice increase in your breathing sensations" during the isoproterenol infusions. Also, to familiarize participants with the experience of the infusion setup and isoproterenol-induced sensations, prior to the scan, participants received two practice bolus infusions (saline and 1 microgram, μg) delivered by the nurse in a separate room near the MRI scanning suite. These infusions followed the same time course as those administered during the scanning session and required participants to provide sensation ratings using a dial. We

© 2022 Teed AR et al. *JAMA Psychiatry.*

administered a 1 μg dose during the practice infusion to avoid a familiarity effect during the subsequent infusion scans, and to ensure that participants received a large enough dose that they were likely to perceive, based on our prior studies[2-4]. Following the scan session participants ate a 1000 Calorie meal.

eMethods 3. Primary and Exploratory Behavioral and Physiological Data Analysis

All statistical tests were performed in R (version 3.5.1; R Core Team, 2016). Linear mixed-effects (LME) regression models were performed using the lme4 package[5], and statistical degrees of freedom were estimated by Satterthwaite's approximation via the lmerTest package[6]. We assigned the reference levels for the group, dose, and epoch variables to be the healthy comparator group, the saline condition, and the baseline epoch, respectively. We estimated the chronotropic dose 25 (CD25) by first performing a within-subject linear regression, by ISO dose, on the maximum average heart rate occurring over any rolling 5 second window during the peak and early recovery periods. We used windowed means as opposed to point estimates to reduce potential measurement error due to outliers, as in our previous studies[7]. To provide a baseline for calculating the CD25, we also obtained rolling 5 second windows for the maximum heartrate occurring during the corresponding 40 second period of a resting state scan run before the task. We then calculated each participant's CD25 estimate using the formula provided in Mills et al.[8], by adding the 25-beat target increase to the resting state value, subtracting the intercept of the linear regression, and dividing the subsequent value by the regression slope. Interoceptive detection was assessed via a chi-square test for independence in R software which counted, for each dose, how many GADs and HCs increased the dial above a threshold of fifteen out of one hundred (this dose detection threshold was chosen to account for inadvertent responses due to the MRI compatible device's sensitivity profile). Differences in cardiorespiratory interoceptive accuracy were assessed by calculating cross-correlations between continuous heart rate and dial ratings for each individual using custom MATLAB script (as in[1]) prior to LME model generation. All physiological and subjective dial responses were down sampled to 1 Hz prior to calculating cross-correlations. We calculated cross correlations for each participant from mean centered dial ratings separately with instantaneous changes in HR and respiratory volume variability (RVV) occurring over the 120 s interval following infusion onset. For each dose, we calculated the cross-correlation with no lag time, the maximum cross-correlation allowing for temporal shifting, and the lag time associated with the maximum cross-correlation. Group and dose differences for each of these coefficients (HR and RVV) were assessed by LME models in an identical manner to the retrospective ratings. Exploratory Pearson correlations for vmPFC cluster PSC and ROI FPC scores with real-time and retrospective cardiorespiratory or anxiety intensity were conducted using the correlation package in R[9] and corrected for multiple comparisons via the Hommel method[10].

eMethods 4. MRI Data Acquisition

MRI scans were acquired in a GE MR750 3T scanner with an 8-channel head coil. Simultaneous EEG recordings were also collected but were not included in the current analysis. We acquired anatomical images via a T1-weighted magnetization-prepared rapid gradient echo (MPRAGE) sequence with sensitivity encoding (SENSE[11]) that lasted 5 min and 40 s. MPRAGE parameters were: 190 axial slices, slice thickness = 0.9 mm, TR/TE = 5/2.012 ms, FOV = 240 x 192 mm^2, matrix size 256 x 256, flip angle = 8º, inversion time = 725 ms, SENSE acceleration R = 2, with a sampling bandwidth of 31.2. A 240 s, single-shot gradient echo planar imaging (EPI) sequence covering the whole-brain was used for each fMRI scan. In this EPI sequence we obtained axial 39 slices, 2.9 mm thick, with no gap and a voxel size of 1.875 x 1.875 x 2.9 mm^3. Additional parameters were TR/TE = 2000/27 ms, FOV = 240 x 240 mm^2, flip angle = 78º, SENSE acceleration R = 2 with a 96 x 96 matrix.

eMethods 5. fMRI Preprocessing

The first 4 EPI volumes were dropped to allow field stabilization. BOLD signal was scaled to percent change from the time-course mean for each voxel. With respect to motion artifacts, poor quality volumes were censored using interpolation if they exceeded 0.3 mm of mean motion or if >20% of voxels were found to be outliers using AFNI's 3dtoutcount. Runs were discarded if >20% of volumes were censored. One run was discarded for each of three HC and two GAD participants. We also checked for potential differences in head motion for each of the infusion conditions and found no group differences.

eMethods 6. Whole-brain fMRI Group Analysis

We conducted whole-brain analysis using AFNI's *3dttest++* program and applied the ETAC (Equitable Thresholding and Clustering) option to estimate significant cluster sizes corresponding to a 5% false positive rate at a $p < 0.001$ voxel-wise threshold[12]. We chose this method to reduce arbitrary judgment in selection of the uncorrected p-value threshold[13].

© 2022 Teed AR et al. *JAMA Psychiatry*.

eMethods 7. Longitudinal time series analysis with regions of interest

Multivariate sparse functional principal component analysis (mSFPCA) was performed to examine how linear and non-linear temporal dynamics of isoproterenol's effects on BOLD response, heart rate, and sensation rating trajectories. This technique characterizes the major modes of smoothed variation around the smoothed mean trajectories as FPC coefficients at the subject level using Hamiltonian Markov chain Monte Carlo (MCMC) resampling of the posterior distribution of model parameters in a Bayesian framework[14]. To assist in functional interpretation of our whole-brain results, we generated separate *post hoc* mSFPCA models for extracted mean PSC from spherical ROIs (5 mm radius) derived from conceptually relevant fMRI meta-analyses reporting clusters in coordinate space near to ours. Seeds for the relevant regions of interest (ROIs) within the vmPFC were obtained from meta-analyses of fMRI studies related to valence processing[15] (x=-3, y=39, z=0), sympathetic autonomic control[16] (x=-4, y=38, z=-12 and x=8, y=42, z=-6), and self-processing[17] (x=2, y=53, z=7). FPC coefficients for HR, dial, and BOLD ROI trajectories were then entered into LME models with group and dose factors to assess how these factors affect those trajectories. The mSFPCA approach we employed uses sets of eigenfunctions that are allowed to correlate across dependent variables but remain independent with regard to each observation within a variable[18]. Using spline basis functions, each individual's response was modeled in a multivariate fashion via a matrix of spline coefficients characterizing the major modes of variation as a set of principal components functions (we chose two) around the smoothed mean trajectory. mSFPCA models were created in R using the MASS[19], Matrix[20], pracma[21], and splines[22] packages. mSFPCA was conducted on HR and dial responses after converting the timescale of these variables to 2 second intervals, with the first four intervals dropped to match the BOLD timeseries given the TR of 2 second and the initial 4 volumes being dropped to allow the magnet to reach a steady-state. We generated mean and PC functions by implementing two internal knots yielding model fits via cubic B-spline basis functions. We ran 1,100 MCMC simulations with 100 of those discarded as burn-in.

© 2022 Teed AR et al. *JAMA Psychiatry*.

eFigure 1. Infusion epochs used in the isoproterenol fMRI analysis.

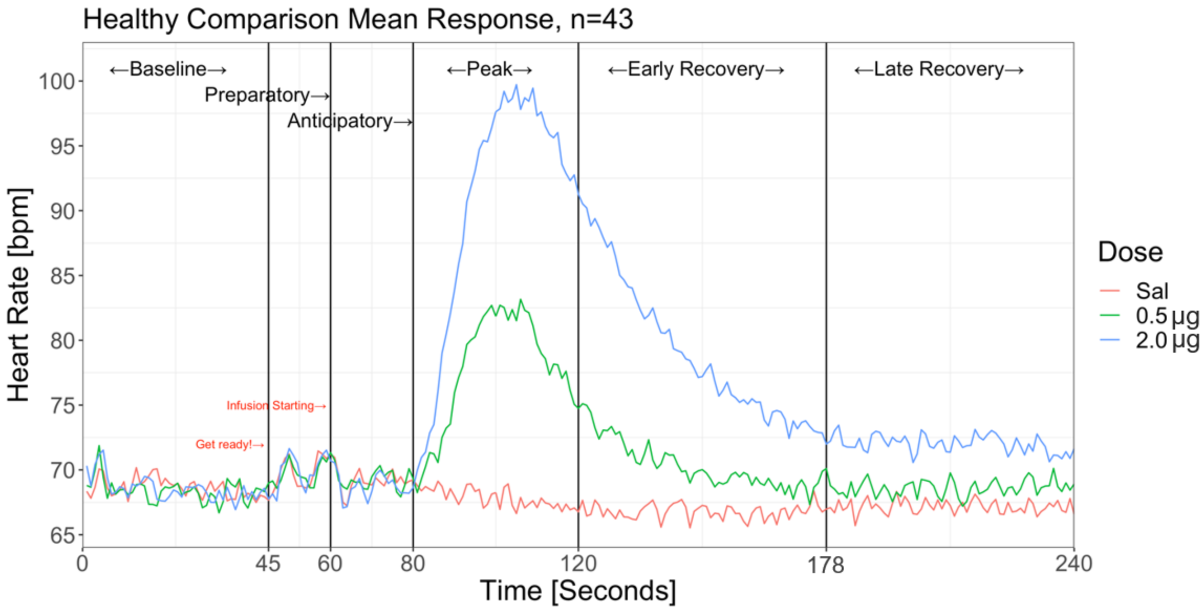

For illustrative purposes, infusion epochs are superimposed on the average time course of heart rate responses during the saline, 0.5 and 2 *µg* isoproterenol infusions in the healthy comparator sample only. The data shown in the figure includes the complete 43 healthy individual sample tested prior to exclusions for head motion (n=5) and age and BMI group matching (n=9). bpm = beats per minute, *µg* = micrograms, Sal = saline.

eFigure 2. Subjective Response Assessment

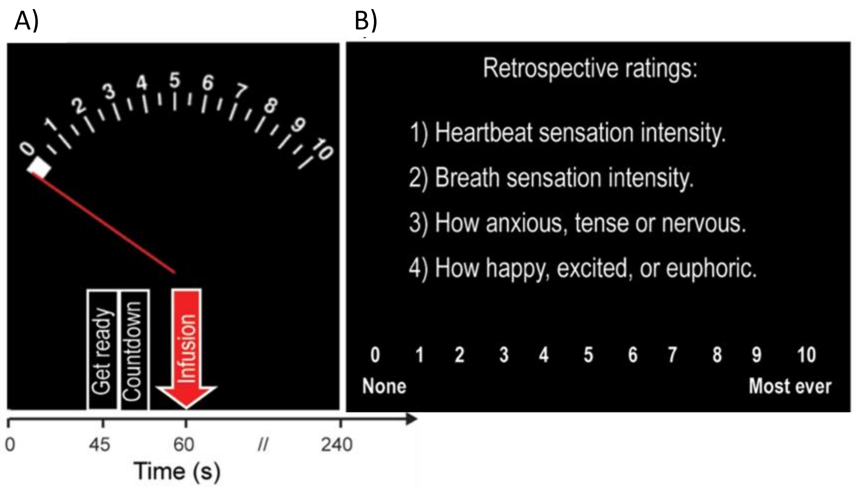

A) Throughout each scan, participants reported their perceived cardiorespiratory sensation intensity ranging using a visual analog scale with a baseline of 0 and maximum of 10 (recorded units of measurement ranged from 0 to 100). B) Immediately after each infusion scan participants provided retrospective ratings of their heartbeat sensation intensity, breathing sensation intensity, anxiety, and arousal felt during the infusions, using an 11-point scale ranging from 0 to 10.

© 2022 Teed AR et al. *JAMA Psychiatry*.

eFigure 3. Respiratory Volume Variability During Isoproterenol Infusion.

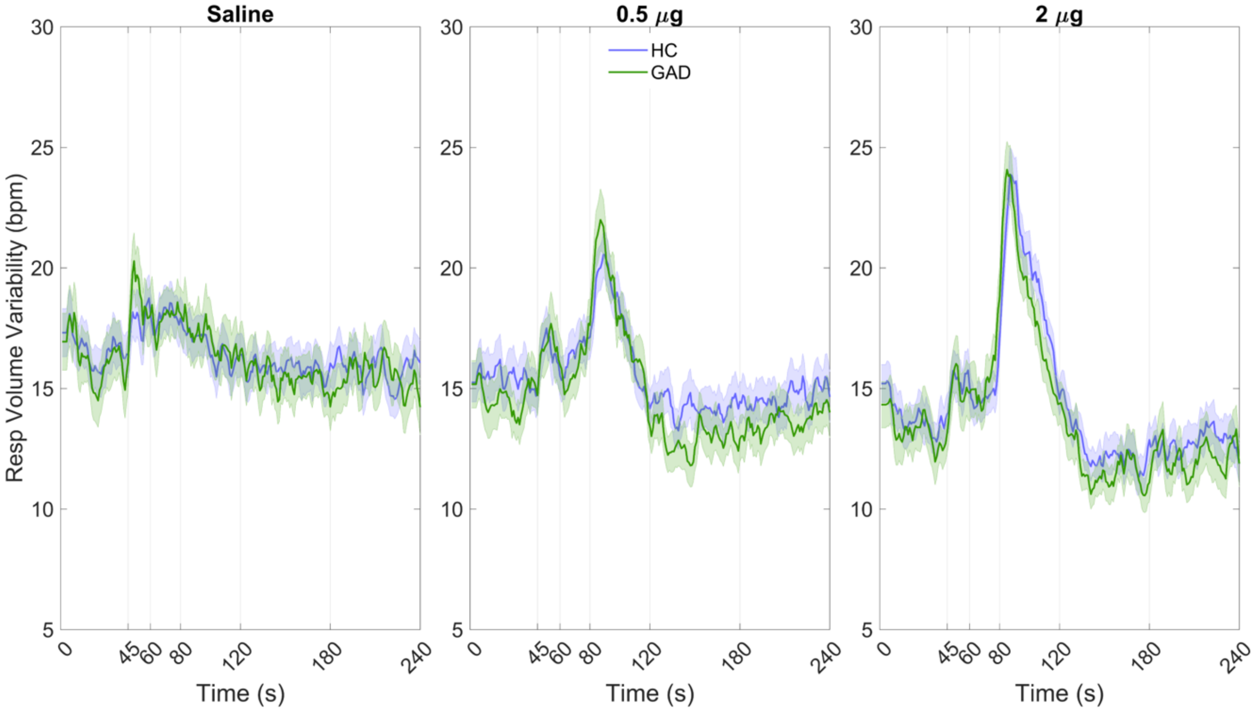

eFigure 4. Interoceptive detection rates.

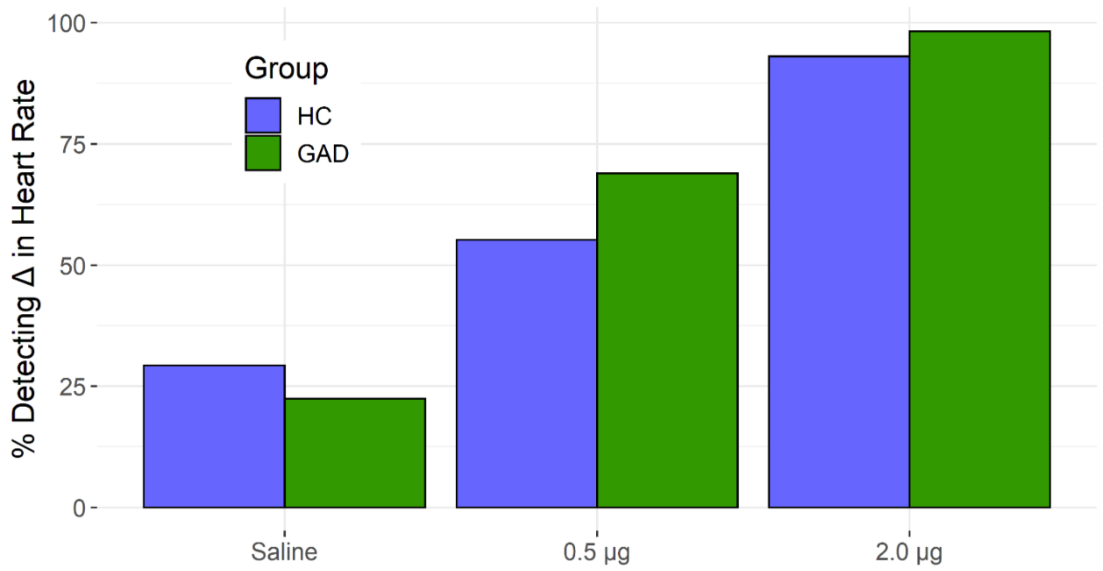

Detection responses were operationalized as the generation of a real-time dial rating of greater than 1.5 on a scale of 10. Interoceptive detection rates increased across dose for both groups, but the groups did not show statistically significant differences in rates of detection.

© 2022 Teed AR et al. *JAMA Psychiatry*.

eFigure 5. Group difference in left parietal cortex activity.

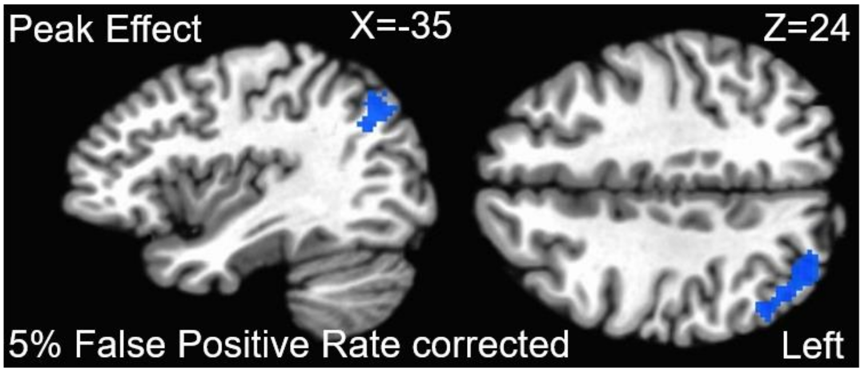

The GAD group showed significant attenuation relative to the HC group at a *p*<0.001 voxel-wise threshold during the peak response period for only the 0.5 µg dose.

eFigure 6. Maximum cross-correlations between heart rate and dial response by dose in unmedicated GAD relative to HC.

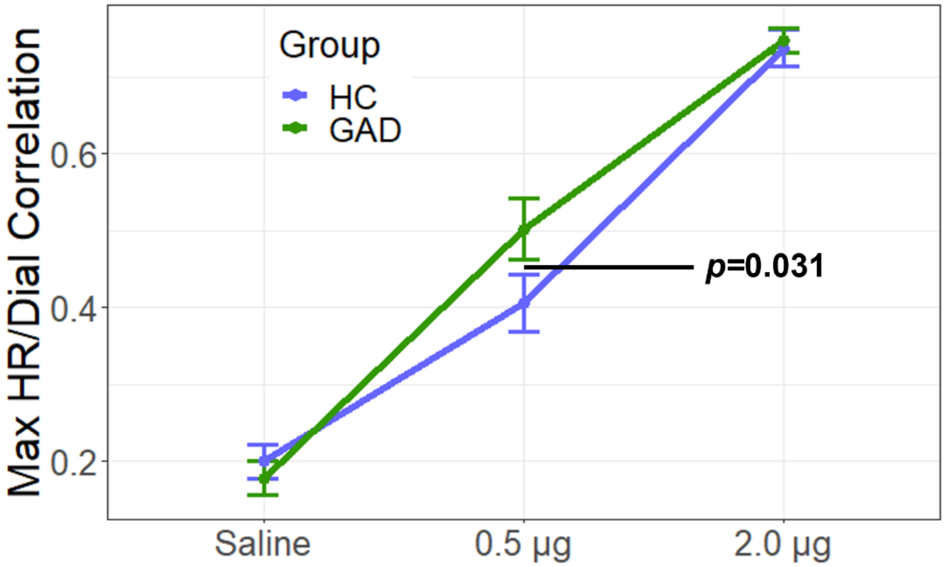

When medicated GAD participants were removed the mean correlation between HR and perceived cardiorespiratory intensity was significantly higher at the 0.5 µg dose in the unmedicated GAD group relative to HCs at the level of the maximum cross-correlation. Error bars = SE.

© 2022 Teed AR et al. *JAMA Psychiatry*.

eFigure 7. Overlap between whole-brain vmPFC cluster and meta-analysis–derived ROIs.

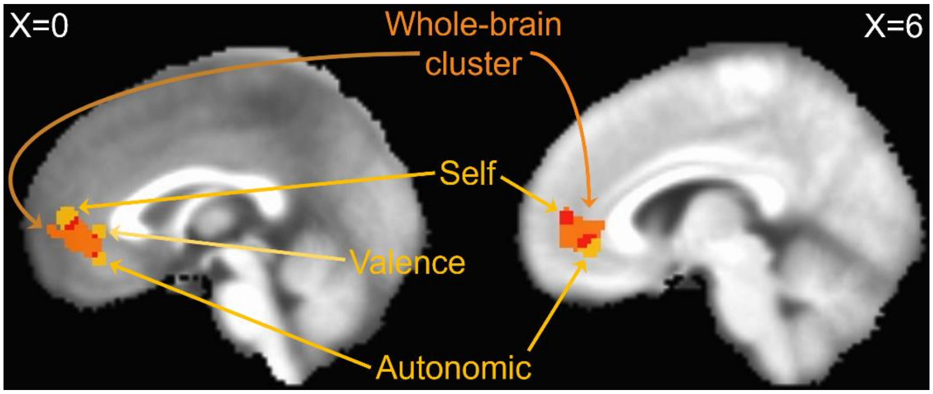

Overlap (red) between the significant whole-brain BOLD vmPFC cluster identified during the peak response period for the 0.5 μg dose (orange) and the meta-analysis–derived functional ROIs (yellow) is shown below.

© 2022 Teed AR et al. *JAMA Psychiatry*.

eFigure 8. Multivariate Sparse Functional Principal Component Analysis (mSFPCA) model fits to raw data by dose and group.

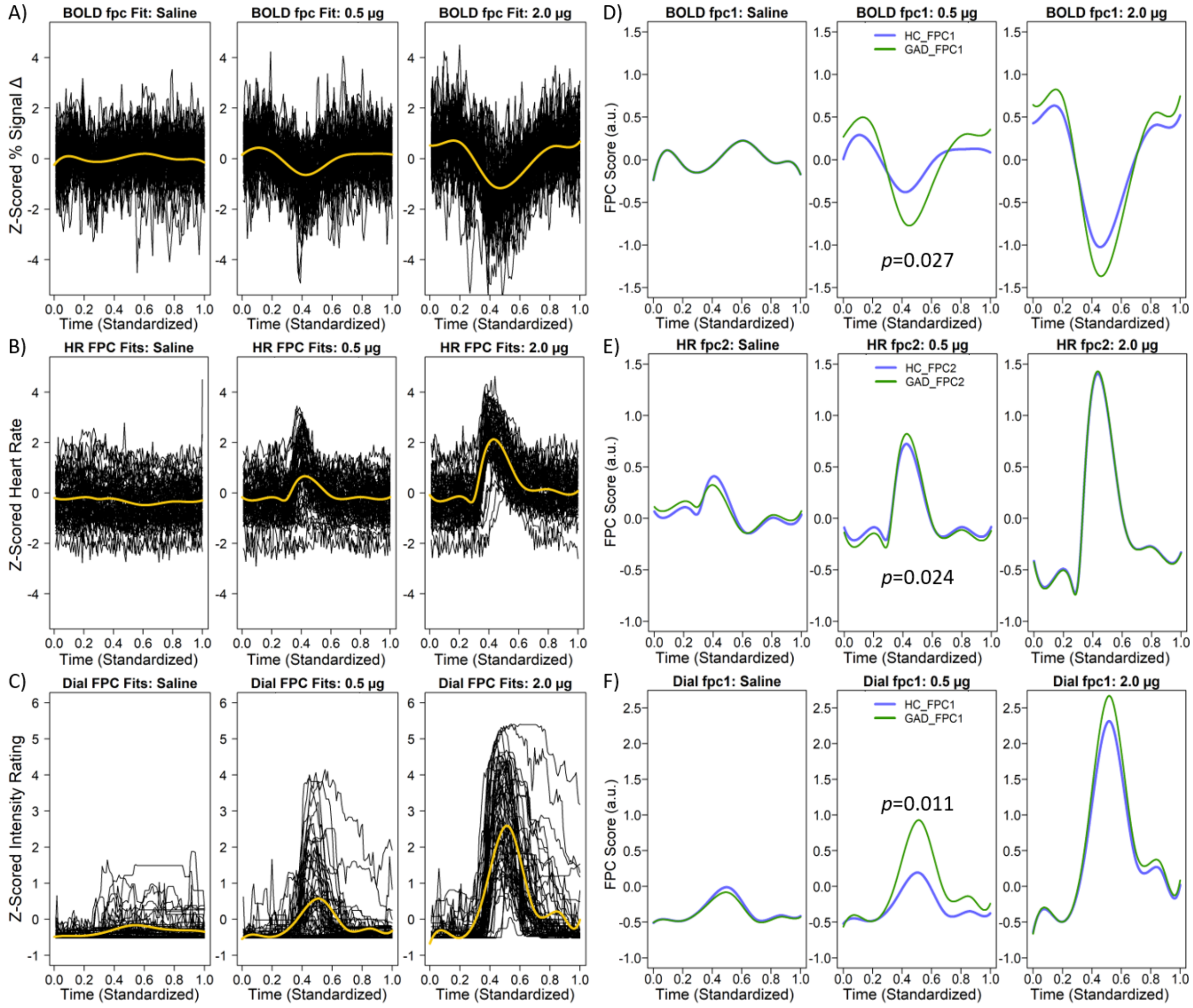

Spaghetti plots of the mfpca fit (yellow lines) to raw data (black lines) for BOLD percent signal of the self-representation vmPFC ROI (A), heart rate (B), and perceived cardiorespiratory intensity (C) are shown to the left. Mean fpc score curves for healthy comparators (blue) and patients with GAD (green) are shown for each of BOLD (D), heart rate E), and perceived intensity (F) to the right.

© 2022 Teed AR et al. *JAMA Psychiatry*.

eFigure 9. VmPFC PSC correlations with cardiorespiratory and subjective variables during the peak effect at 0.5*μg* isoproterenol

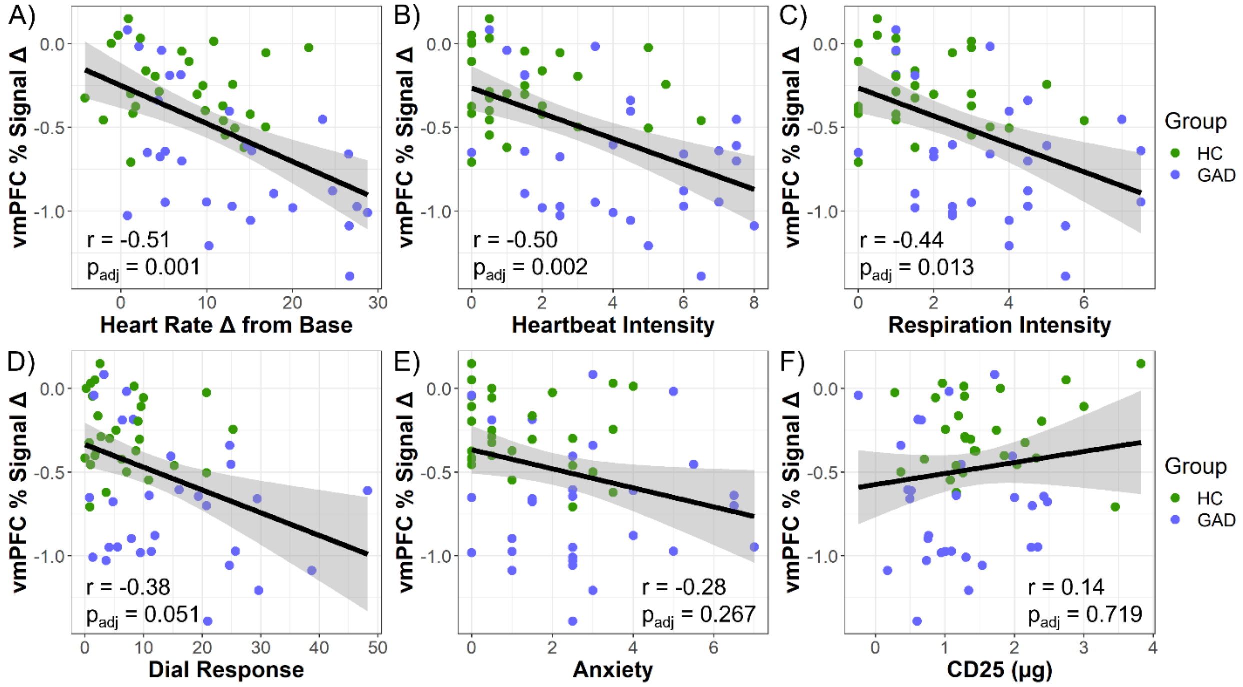

Scatterplots with correlation slope line and standard deviation band for relationships between percent signal change extracted from the whole-brain vmPFC selected cardiorespiratory and self-report variables. After Bonferroni-Holm correction for multiple comparisons, significant inverse relationships were seen between PSC and A) change in heart rate from pre-infusion baseline as well as post-infusion self-report of both B) beartbeat intensity and C) respiratory intensity. Negative PSC relationships not surviving Bonferroni-Holm correction were seen for D) perceived cardiorespiratory intensity, though this remained a trend, and E) self-reported anxiety. The relationship with F) CD25 was not significant. CD25 = chronotropic dose 25

© 2022 Teed AR et al. *JAMA Psychiatry*.

eResults 1. Respiratory Volume Variability (RVV)

We observed dose-related increases in RVV during the peak period but no interaction with group (eFigure 3 and eTable 5), suggesting that group differences in perceived interoceptive intensity were driven preferentially by cardiac signals.

eResults 2. Interoceptive Awareness: Detection and Accuracy Measures

A chi-square test to assess group differences in detection rate of cardiorespiratory changes via increased dial ratings was not significant ($\chi(1)=2.54$, $p=0.111$; (eFigure 4). Nor were there clear differences in interoceptive accuracy, as assessed via the cross-correlation of HR with dial responses. The maximum cross-correlation was the only metric showing a trend toward increased accuracy for GADs during a combined peak + early recovery period at 0.5µg ($b=0.09$, SE=0.05, $t(281.53)=1.79$, $p=0.075$; eTables 6 and 7).

eResults 3. Reanalysis of Primary Outcomes with Medicated Participants Removed

A reanalysis with the six medicated patients removed revealed a significant difference in age between groups (t(38.28), p<0.047) with GADs being older (M=27.96 years) than HCs (M=24.38 years); age was included as a covariate in the subsequent analyses (as it was in the original models). We observed an additional significant result of higher HR for those with GAD compared to HCs at the 2mcg dose during the peak period (β=3.76, t(1475.93)=2.11, p=0.035, 95%CI[0.30, 7.22]). The dial response interaction of GAD group by 0.5mcg dose became non-significant during the peak epoch (β=5.42, t(1476.73)=1.57, p=0.118, 95%CI[-1.30, 12.15]) but remained significant during early recovery (β=7.62, t(1476.77)=2.00, p=0.028, 95%CI[0.89, 14.36]). Finally, the interoceptive accuracy cross-correlation interaction of GAD group by 0.5µg dose became significant (β=0.12, t(252.03)=2.17, p=0.031, 95%CI[0.01, 0.22]; eFigure 6). Results relating to BMI, CD25, and all retrospective ratings were unchanged with respect to statistical significance and nearly identical to the previous analysis.

eResults 4. Longitudinal Time-Series Analysis

The mSFPCA LME revealed an effect of group at 0.5µg for the self-processing ROI (β=0.23, $t(112)=2.24$, $p=0.027$, 95%CI[0.03, 0.44]) and a trend at 2.0µg (β=0.19, $t(112)=1.85$, $p=0.067$, 95%CI[-0.01, 0.40]) on FPC1 (Figure 3D, eFigure 8D and eTable 8). For the valence ROI LME, only dose effects were observed for FPC1 but a group effect was seen for FPC2 (β=0.11, $t(56)=2.09$, $p=0.038$, 95%CI[0.01, 0.22]). The sympathetic autonomic ROI LME showed a trend for a group by dose interaction at 0.5µg on FPC1 (β=0.25, $t(112)=1.85$, $p=0.067$, 95%CI[-0.01, 0.51]). When PSC values for this ROI were entered in an epoch-based LME, a significant decrease for GAD was seen at 0.5µg during early recovery (β=-0.58, $t(840)=-2.17$, $p=0.030$, 95%CI[-1.09, -0.06]) (eTable 9; eTables 10 and 11 for other ROIs). Group interactions reflecting differences in HR on FPC2 (β=-0.11, $t(112)=-2.28$, $p=0.025$, 95%CI[-0.20, -0.02]) and cardiorespiratory dial ratings on FPC1 (β=0.39, $t(112)=2.79$, $p=0.006$, 95%CI[0.12, -0.67]) were also observed at the 0.5µg dose. In each model, dose effects were again seen on the trajectory of HR and dial ratings (eTable 12).

© 2022 Teed AR et al. *JAMA Psychiatry*.

eTable 1. Diagnostic comorbidities and psychotropic medication status of generalized anxiety disorder and healthy comparator participants.

	GAD	HC
**Psychotropic medication, N (%)[a]**	6 (21)	0
**Comorbid Diagnoses, N (%)[b]**		
Major depressive disorder (recurrent)	15 (52)	0
Major depressive disorder (partial remission)	9 (31)	0
Social anxiety disorder	14 (48)	0
Posttraumatic stress disorder	3 (10)	0
**Race/Ethnicity, N (%)[c]**		
Black or African American	2(7)	2(7)
American Indian or Alaskan Native	1(3)	1(3)
Asian	4(14)	5(17)[d]
Hispanic or Latino	3(10)	3(10)
White	19(66)	18(62)
Other	0	0[e]

[a] 3 participants reported selective serotonin reuptake inhibitor use (sertraline, paroxetine), 2 reported selective norepinephrine reuptake inhibitor use (bupropion, escitalopram), 1 reported taking both classes of reuptake inhibitor, 1 reported occasional as needed use of a short-acting beta-blocker (propranolol) but abstained for at least one day prior to scanning and 1 reported medicinal tetrahydrocannabinol (THC) use but tested negative on the day of scanning. No participants were taking scheduled benzodiazepines or barbiturates.

[b] For brevity, only comorbid diagnoses with frequency > 5% are listed.

[c] Racial and ethnic categories defined as per the most recent revisions of the federal Office of Management and Budget Directive 15.

[d] One healthy comparator responded as being of Vietnamese and Hispanic ethnicity.

[e] One healthy comparator refused to report ethnicity.

© 2022 Teed AR et al. *JAMA Psychiatry*.

eTable 2. LME for Heart Rate by Group and Dose for Selected Isoproterenol Time-course Epochs.

Factor	Estimate	Std. Err.	DF	t-Stat	p-Value	Sig.
(Intercept)	*43.60*	*13.79*	*54.15*	*3.16*	*0.003*	**
Period Ant	-0.04	0.84	1650.04	-0.05	0.959	
Period Peak	-0.86	0.84	1650.04	-1.02	0.308	
Period Early	*-1.74*	*0.84*	*1650.04*	*-2.06*	*0.039*	*
Period Late	-1.59	0.84	1650.04	-1.89	0.059	^
Dose 0.5 µg	0.24	0.84	1650.04	0.29	0.772	
Dose 2.0 µg	-0.02	0.84	1650.04	-0.02	0.984	
Group GAD	27.66	14.03	56.20	1.97	0.054	^
Age	0.26	0.38	53.96	0.71	0.483	
BMI	0.69	0.60	53.96	1.15	0.257	
Ant X 0.5 µg	-0.12	1.19	1650.04	-0.10	0.921	
Peak X 0.5 µg	*8.23*	*1.19*	*1650.04*	*6.91*	*<0.001*	***
Early X 0.5 µg	2.13	1.19	1650.04	1.79	0.075	^
Late X 0.5 µg	1.07	1.19	1650.04	0.90	0.369	
Ant X 2.0 µg	-0.51	1.19	1650.04	-0.43	0.670	
Peak X 2.0 µg	*22.68*	*1.19*	*1650.04*	*19.04*	*<0.001*	***
Early X 2.0 µg	*13.79*	*1.19*	*1650.04*	*11.57*	*<0.001*	***
Late X 2.0 µg	*5.86*	*1.19*	*1650.04*	*4.92*	*<0.001*	***
Ant X GAD	0.68	1.19	1650.04	0.57	0.568	
Peak X GAD	0.33	1.19	1650.04	0.28	0.782	
Early X GAD	0.14	1.19	1650.04	0.12	0.908	
Late X GAD	0.59	1.19	1650.04	0.49	0.623	
0.5 µg X GAD	-0.12	1.19	1650.04	-0.10	0.921	
2.0 µg X GAD	-0.30	1.19	1650.04	-0.25	0.801	
GAD X Age	-0.30	0.38	55.16	-0.80	0.427	
GAD X BMI	-0.73	0.61	55.15	-1.21	0.231	
Ant X 0.5 µg X GAD	0.32	1.68	1650.04	0.19	0.847	
Peak X 0.5 µg X GAD	*5.34*	*1.68*	*1650.04*	*3.17*	*0.002*	**
Early X 0.5 µg X GAD	2.02	1.69	1650.04	1.20	0.232	
Late X 0.5 µg X GAD	0.71	1.69	1650.04	0.42	0.672	
Ant X 2.0 µg X GAD	1.09	1.68	1650.04	0.65	0.519	
Peak X 2.0 µg X GAD	3.18	1.68	1650.04	1.89	0.060	^
Early X 2.0 µg X GAD	-0.95	1.69	1650.04	-0.56	0.573	
Late X 2.0 µg X GAD	-1.46	1.69	1650.04	-0.87	0.385	

*Note: Sig. = significance. Coded as *** = p<0.001, ** = p<0.01, * = p<0.05, ^ = p<0.1*

© 2022 Teed AR et al. *JAMA Psychiatry*.

eTable 3. LME for Continuous Cardiorespiratory Dial Ratings by Group and Dose for Selected Isoproterenol Time-course Epochs.

Factor	Estimate	Std. Err.	DF	t-Stat	p-Value	Sig.
(Intercept)	8.97	7.18	56.41	1.25	0.217	
Period Ant	0.46	1.63	1650.55	0.28	0.776	
Period Peak	2.51	1.63	1650.55	1.54	0.123	
Period Early	*3.75*	*1.63*	*1650.55*	*2.30*	*0.021*	*
Period Late	2.81	1.63	1650.55	1.73	0.084	^
Dose 0.5 µg	-0.14	1.63	1650.55	-0.09	0.931	
Dose 2.0 µg	0.51	1.63	1650.55	0.31	0.754	
Group GAD	-13.75	7.96	75.44	-1.73	0.088	^
Age	-0.19	0.19	53.74	-0.98	0.331	
BMI	-0.14	0.31	53.74	-0.45	0.655	
Ant X 0.5 µg	0.14	2.30	1650.55	0.06	0.952	
Peak X 0.5 µg	3.91	2.30	1650.55	1.70	0.090	^
Early X 0.5 µg	1.34	2.30	1650.55	0.58	0.560	
Late X 0.5 µg	-1.20	2.30	1650.55	-0.52	0.602	
Ant X 2.0 µg	0.39	2.30	1650.55	0.17	0.866	
Peak X 2.0 µg	*25.27*	*2.30*	*1650.55*	*10.97*	*<0.001*	***
Early X 2.0 µg	*27.14*	*2.30*	*1650.55*	*11.79*	*<0.001*	***
Late X 2.0 µg	*4.57*	*2.30*	*1650.55*	*1.99*	*0.047*	*
Ant X GAD	0.24	2.30	1650.55	0.11	0.916	
Peak X GAD	-0.18	2.30	1650.55	-0.08	0.938	
Early X GAD	-1.53	2.31	1650.61	-0.66	0.508	
Late X GAD	0.01	2.31	1650.61	0.01	0.995	
0.5 µg X GAD	0.45	2.30	1650.55	0.20	0.844	
2.0 µg X GAD	0.24	2.30	1650.55	0.11	0.917	
GAD X Age	0.30	0.21	65.82	1.47	0.148	
GAD X BMI	0.25	0.33	64.56	0.76	0.450	
Ant X 0.5 µg X GAD	0.41	3.26	1650.55	0.13	0.901	
Peak X 0.5 µg X GAD	*8.38*	*3.26*	*1650.55*	*2.57*	*0.010*	*
Early X 0.5 µg X GAD	*9.11*	*3.26*	*1650.58*	*2.80*	*0.005*	**
Late X 0.5 µg X GAD	1.88	3.26	1650.58	0.58	0.564	
Ant X 2.0 µg X GAD	0.44	3.26	1650.55	0.13	0.893	
Peak X 2.0 µg X GAD	6.06	3.26	1650.55	1.86	0.063	^
Early X 2.0 µg X GAD	3.36	3.26	1650.58	1.03	0.303	
Late X 2.0 µg X GAD	-1.19	3.26	1650.58	-0.37	0.714	

*Note: Sig. = significance. Coded as *** = p<0.001, ** = p<0.01, * = p<0.05, ^ = p<0.1*

© 2022 Teed AR et al. *JAMA Psychiatry*.

eTable 4. LME for Retrospective Ratings by Group and Dose.

Factor	Estimate	Std. Err.	DF	t-Stat	p-Value	Sig.
**Heartbeat Intensity**						
(Intercept)	*5.27*	*1.89*	*52.68*	*2.79*	*0.007*	**
Dose 0.5 µg	*0.79*	*0.31*	*278.98*	*2.54*	*0.012*	*
Dose 2.0 µg	*5.35*	*0.31*	*279.27*	*17.12*	*<0.001*	***
Group GAD	-3.04	2.40	52.88	-1.27	0.210	
Age	-0.05	0.05	52.04	-1.05	0.297	
BMI	-0.13	0.08	51.86	-1.57	0.122	
0.5 µg X GAD	*2.21*	*0.44*	*278.81*	*5.04*	*<0.001*	***
2.0 µg X GAD	0.50	0.44	278.96	1.14	0.257	
GAD X Age	0.05	0.06	51.78	0.73	0.469	
GAD X BMI	0.09	0.10	51.83	0.97	0.336	
**Respiratory Intensity**						
(Intercept)	*4.40*	*1.88*	*52.39*	*2.34*	*0.023*	*
Dose 0.5 µg	*0.85*	*0.29*	*276.49*	*2.90*	*0.004*	**
Dose 2.0 µg	*4.27*	*0.29*	*275.76*	*14.86*	*<0.001*	***
Group GAD	-2.46	2.39	52.52	-1.03	0.309	
Age	-0.03	0.05	51.68	-0.58	0.566	
BMI	-0.11	0.08	52.08	-1.36	0.181	
0.5 µg X GAD	*1.51*	*0.41*	*276.00*	*3.70*	*<0.001*	***
2.0 µg X GAD	0.48	0.41	275.62	1.18	0.238	
GAD X Age	0.04	0.06	51.50	0.67	0.506	
GAD X BMI	0.07	0.10	51.88	0.67	0.507	
**Anxiety**						
(Intercept)	2.43	2.01	52.71	1.21	0.231	
Dose 0.5 µg	0.47	0.26	281.50	1.81	0.072	.
Dose 2.0 µg	1.63	0.26	281.09	6.34	<0.001	***
Group GAD	0.19	2.54	52.80	0.07	0.942	
Age	-0.02	0.05	51.97	-0.31	0.756	
BMI	-0.05	0.09	52.83	-0.54	0.593	
0.5 µg X GAD	*1.04*	*0.37*	*281.32*	*2.84*	*0.005*	**
2.0 µg X GAD	*1.22*	*0.36*	*281.11*	*3.36*	*0.001*	***
GAD X Age	0.06	0.07	51.95	0.96	0.341	
GAD X BMI	-0.05	0.10	52.60	-0.52	0.604	
**Excitement**						
(Intercept)	4.09	2.21	51.91	1.85	0.071	^
Dose 0.5 µg	-0.02	0.18	256.70	-0.10	0.922	
Dose 2.0 µg	0.14	0.18	256.62	0.76	0.450	
Group GAD	-3.20	2.81	51.99	-1.14	0.260	
Age	0.35	0.26	256.69	1.37	0.173	
BMI	0.18	0.26	256.89	0.69	0.489	
0.5 µg X GAD	0.04	0.06	51.67	0.70	0.486	
2.0 µg X GAD	0.07	0.04	51.80	1.57	0.123	
GAD X Age	-0.15	0.10	52.26	-1.55	0.128	
GAD X BMI	-0.07	0.06	51.80	-1.19	0.239	

*Note: Sig. = significance. Coded as *** = p<0.001, ** = p<0.01, * = p<0.05, ^ = p<0.1*

© 2022 Teed AR et al. *JAMA Psychiatry*.

eTable 5. LME for Respiratory Volume Variability by Group and Dose for Selected Isoproterenol Time-course Epochs.

Factor	Estimate	Std. Err.	DF	t-Stat	p-Value	Sig.
(Intercept)	*15.59*	*3.87*	*54.63*	*4.03*	*<0.001*	***
Period Ant	*1.37*	*0.59*	*1649.87*	*2.32*	*0.021*	*
Period Peak	0.23	0.59	1649.87	0.39	0.697	
Period Early	-0.65	0.59	1649.87	-1.10	0.270	
Period Late	-0.81	0.59	1649.87	-1.38	0.169	
Dose 0.5 µg	-1.14	0.59	1649.87	-1.94	0.052	^
Dose 2.0 µg	*-3.07*	*0.59*	*1649.87*	*-5.21*	*<0.001*	***
Group GAD	1.16	4.11	65.40	0.28	0.778	
Age	0.20	0.10	53.46	1.89	0.065	^
BMI	-0.16	0.17	53.46	-0.94	0.352	
Ant X 0.5 µg	0.13	0.83	1649.87	0.16	0.872	
Peak X 0.5 µg	*2.77*	*0.83*	*1649.87*	*3.33*	*<0.001*	***
Early X 0.5 µg	-0.76	0.83	1649.87	-0.92	0.360	
Late X 0.5 µg	-0.07	0.83	1649.87	-0.08	0.935	
Ant X 2.0 µg	-0.30	0.83	1649.87	-0.36	0.719	
Peak X 2.0 µg	*6.18*	*0.83*	*1649.87*	*7.41*	*<0.001*	***
Early X 2.0 µg	-0.42	0.83	1649.87	-0.51	0.610	
Late X 2.0 µg	-0.29	0.83	1649.87	-0.35	0.727	
Ant X GAD	0.34	0.83	1649.87	0.41	0.681	
Peak X GAD	0.19	0.83	1649.87	0.22	0.823	
Early X GAD	0.03	0.84	1649.89	0.04	0.970	
Late X GAD	0.40	0.84	1649.89	0.48	0.631	
0.5 µg X GAD	-0.33	0.83	1649.87	-0.40	0.688	
2.0 µg X GAD	0.32	0.83	1649.87	0.38	0.702	
GAD X Age	-0.21	0.11	59.97	-1.93	0.058	^
GAD X BMI	0.15	0.17	59.63	0.89	0.375	
Ant X 0.5 µg X GAD	-0.77	1.18	1649.87	-0.66	0.512	
Peak X 0.5 µg X GAD	0.15	1.18	1649.87	0.13	0.900	
Early X 0.5 µg X GAD	-0.43	1.18	1649.88	-0.36	0.718	
Late X 0.5 µg X GAD	-0.59	1.18	1649.88	-0.50	0.614	
Ant X 2.0 µg X GAD	0.33	1.18	1649.87	0.28	0.780	
Peak X 2.0 µg X GAD	-1.24	1.18	1649.87	-1.05	0.293	
Early X 2.0 µg X GAD	-0.60	1.18	1649.88	-0.51	0.612	
Late X 2.0 µg X GAD	-0.67	1.18	1649.88	-0.57	0.572	

*Note: Sig. = significance. Coded as *** = p<0.001, ** = p<0.01, * = p<0.05, ^ = p<0.1*

© 2022 Teed AR et al. *JAMA Psychiatry.*

eTable 6. LME for Cross Correlations (CC) Between Real-time Dial Responses and Heart Rate.

Factor	Estimate	Std. Err.	DF	t-Stat	p-Value	Sig.
**Zero-Lag CC**						
(Intercept)	0.09	0.14	332.08	0.62	0.538	
Dose 0.5 µg	*0.17*	*0.04*	*280.94*	*4.44*	*<0.001*	***
Dose 2.0 µg	*0.63*	*0.04*	*280.94*	*16.36*	*<0.001*	***
Group GAD	-0.06	0.18	332.56	-0.36	0.721	
Age	0.00	0.00	313.91	-0.83	0.407	
BMI	0.00	0.01	301.96	-0.44	0.664	
0.5 µg X GAD	0.05	0.05	281.12	0.88	0.380	
2.0 µg X GAD	-0.07	0.05	281.12	-1.34	0.181	
GAD X Age	0.00	0.00	332.12	0.82	0.411	
GAD X BMI	0.00	0.01	318.40	0.02	0.983	
**Max CC**						
(Intercept)	0.25	0.13	336.37	1.95	0.052	^
Dose 0.5 µg	*0.21*	*0.04*	*281.27*	*5.69*	*<0.001*	***
Dose 2.0 µg	*0.54*	*0.04*	*281.27*	*14.87*	*<0.001*	***
Group GAD	-0.06	0.16	336.20	-0.39	0.700	
Age	0.00	0.00	325.11	1.07	0.286	
BMI	-0.01	0.01	310.95	-1.09	0.276	
0.5 µg X GAD	0.09	0.05	281.53	1.79	0.075	^
2.0 µg X GAD	-0.01	0.05	281.53	-0.19	0.846	
GAD X Age	0.00	0.00	337.00	-0.82	0.416	
GAD X BMI	0.01	0.01	328.38	1.01	0.311	
**Lag-time at Max CC**						
(Intercept)	9.55	20.32	279.00	0.47	0.639	
Dose 0.5 µg	*-14.04*	*6.37*	*279.00*	*-2.20*	*0.028*	*
Dose 2.0 µg	*-17.47*	*6.05*	*279.00*	*-2.89*	*0.004*	**
Group GAD	-15.44	25.67	279.00	-0.60	0.548	
Age	-0.29	0.55	279.00	-0.54	0.592	
BMI	0.22	0.87	279.00	0.25	0.800	
0.5 µg X GAD	5.57	8.83	279.00	0.63	0.529	
2.0 µg X GAD	13.33	8.47	279.00	1.57	0.117	
GAD X Age	0.30	0.66	279.00	0.46	0.649	
GAD X BMI	-0.22	1.03	279.00	-0.21	0.831	

*Note: Sig. = significance. Coded as *** = p<0.001, ** = p<0.01, * = p<0.05, ^ = p<0.1*

© 2022 Teed AR et al. *JAMA Psychiatry*.

eTable 7. LME for Cross Correlations (CC) Between Continuous Dial Responses and Respiratory Volume Variability.

Factor	Estimate	Std. Err.	DF	t-Stat	p-Value	Sig.
**Zero-Lag CC**						
(Intercept)	0.00	0.16	331.09	0.02	0.982	
Dose 0.5 µg	0.03	0.04	281.73	0.66	0.511	
Dose 2.0 µg	0.00	0.04	281.73	-0.02	0.981	
Group GAD	0.03	0.20	331.89	0.17	0.866	
Age	0.00	0.00	311.58	0.16	0.870	
BMI	0.00	0.01	300.58	-0.58	0.564	
0.5 µg X GAD	-0.06	0.06	281.89	-1.06	0.292	
2.0 µg X GAD	-0.03	0.06	281.89	-0.45	0.650	
GAD X Age	0.00	0.01	329.85	0.29	0.774	
GAD X BMI	0.00	0.01	316.10	-0.14	0.888	
**Max CC**						
(Intercept)	*0.35*	*0.12*	*334.24*	*2.83*	*0.005*	**
Dose 0.5 µg	*0.10*	*0.03*	*281.77*	*3.04*	*0.003*	**
Dose 2.0 µg	*0.23*	*0.03*	*281.77*	*6.90*	*<0.001*	***
Group GAD	-0.26	0.16	334.28	-1.66	0.099	^
Age	0.00	0.00	319.23	0.56	0.574	
BMI	-0.01	0.01	306.22	-1.10	0.273	
0.5 µg X GAD	0.06	0.05	281.98	1.21	0.229	
2.0 µg X GAD	0.03	0.05	281.98	0.69	0.488	
GAD X Age	0.00	0.00	335.41	0.11	0.913	
GAD X BMI	0.01	0.01	323.27	1.70	0.091	^
**Lag-time at Max CC**						
(Intercept)	12.29	28.80	278.92	0.43	0.670	
Dose 0.5 µg	-4.79	8.73	242.07	-0.55	0.584	
Dose 2.0 µg	-3.46	8.30	242.55	-0.42	0.677	
Group GAD	-7.19	36.34	278.80	-0.20	0.843	
Age	-0.63	0.77	274.03	-0.82	0.415	
BMI	-0.07	1.21	262.65	-0.05	0.957	
0.5 µg X GAD	-0.38	12.11	242.32	-0.03	0.975	
2.0 µg X GAD	-3.42	11.61	242.09	-0.30	0.768	
GAD X Age	0.55	0.94	278.24	0.59	0.558	
GAD X BMI	-0.44	1.44	274.14	-0.31	0.759	

*Note: Sig. = significance. Coded as *** = p<0.001, ** = p<0.01, * = p<0.05, ^ = p<0.1*

© 2022 Teed AR et al. *JAMA Psychiatry*.

eTable 8. LMEs for FPC Score Reflecting Signal Change Timeseries for Meta-analysis–derived vmPFC ROIs.

Factor	Value	Std. Err.	DF	t-value	p-value	Sig.
**Self ROI: Functional Principal Component 1**						
(Intercept)	-0.35	0.06	146.00	-5.64	<.001	***
Group GAD	0.00	0.09	146.00	0.01	0.944	
Dose 0.5 µg	*0.22*	*0.07*	*112.00*	*2.99*	*0.004*	**
Dose 2.0 µg	*0.60*	*0.07*	*112.00*	*8.11*	*<.001*	***
0.5 µg X GAD	*0.23*	*0.10*	*112.00*	*2.24*	*0.027*	*
2.0 µg X GAD	0.19	0.10	112.00	1.85	0.067	^
**Self ROI: Functional Principal Component 2**						
(Intercept)	-0.06	0.04	162.85	-1.49	0.138	
Group GAD	0.09	0.06	162.85	1.52	0.129	
Dose 0.5 µg	0.10	0.05	112.00	1.85	0.067	^
Dose 2.0 µg	0.03	0.05	112.00	0.52	0.607	
0.5 µg X GAD	-0.05	0.08	112.00	-0.66	0.509	
2.0 µg X GAD	-0.11	0.08	112.00	-1.41	0.160	
**Valence ROI: Functional Principal Component 1**						
(Intercept)	-0.38	0.08	138.39	-4.93	<.001	***
Group GAD	0.07	0.11	138.39	0.63	0.528	
Dose 0.5 µg	*0.21*	*0.09*	*112.00*	*2.34*	*0.021*	*
Dose 2.0 µg	*0.69*	*0.09*	*112.00*	*7.73*	*<.001*	***
0.5 µg X GAD	0.16	0.13	112.00	1.31	0.194	
2.0 µg X GAD	0.12	0.13	112.00	0.96	0.341	
**Valence ROI: Functional Principal Component 2**						
(Intercept)	-0.01	0.04	138.46	-0.28	0.779	
Group GAD	*0.12*	*0.06*	*138.46*	*2.09*	*0.038*	*
Dose 0.5 µg	0.03	0.05	112.00	0.55	0.584	
Dose 2.0 µg	*-0.11*	*0.05*	*112.00*	*-2.21*	*0.029*	*
0.5 µg X GAD	-0.10	0.06	112.00	-1.19	0.237	
2.0 µg X GAD	-0.07	0.06	112.00	-0.88	0.382	
**Autonomic ROI: Functional Principal Component 1**						
(Intercept)	-0.41	0.08	144.63	-5.10	<.001	***
Group GAD	-0.00	0.11	144.63	-0.03	0.976	
Dose 0.5 µg	0.16	0.10	112.00	1.67	0.097	
Dose 2.0 µg	*0.88*	*0.10*	*112.00*	*9.27*	*<.001*	***
0.5 µg X GAD	0.25	0.13	112.00	1.85	0.067	^
2.0 µg X GAD	0.09	0.13	112.00	0.69	0.494	
**Autonomic ROI: Functional Principal Component 2**						
(Intercept)	-0.04	0.05	133.05	-0.86	0.394	
Group GAD	0.11	0.06	133.05	1.67	0.097	^
Dose 0.5 µg	*0.09*	*0.05*	*112.00*	*1.77*	*0.080*	^
Dose 2.0 µg	*-0.11*	*0.05*	*112.00*	*-2.13*	*0.036*	*
0.5 µg X GAD	-0.09	0.07	112.00	-1.22	0.224	
2.0 µg X GAD	0.04	0.07	112.00	0.58	0.560	

*Note: Sig. = significance. Coded as *** = p<0.001, ** = p<0.01, * = p<0.05, ^ = p<0.1*

© 2022 Teed AR et al. *JAMA Psychiatry*.

eTable 9. LME for BOLD PSC for the Meta-analysis–derived Autonomic vmPFC ROI.

Factor	Value	Std. Err.	DF	t-stat	p-value	Sig.
(Intercept)	0.10	0.22	836.00	0.45	0.653	
Early	-0.26	0.13	836.00	-1.93	0.054	^
Late	-0.15	0.13	836.00	-1.11	0.268	
Peak	-0.50	0.13	836.00	-3.78	*<0.001*	***
Ant	-0.50	0.13	836.00	-3.76	*<0.001*	***
Dose 0.5 µg	0.12	0.13	836.00	0.87	0.387	
Dose 2.0 µg	0.60	0.13	836.00	4.55	*<0.001*	***
Group GAD	0.09	0.28	836.00	0.31	0.758	
Age	0.00	0.01	836.00	0.20	0.841	
BMI	0.00	0.01	836.00	0.52	0.601	
Early X 0.5 µg	-0.13	0.19	836.00	-0.68	0.495	
Late X 0.5 µg	-0.03	0.19	836.00	-0.16	0.871	
Peak X 0.5 µg	*-0.41*	*0.19*	*836.00*	*-2.20*	*0.028*	*
Ant X 0.5 µg	-0.04	0.19	836.00	-0.21	0.835	
Early X 2.0 µg	-1.62	0.19	836.00	-8.63	*<0.001*	***
Late X 2.0 µg	-0.02	0.19	836.00	-0.12	0.909	
Peak X 2.0 µg	-1.32	0.19	836.00	-7.02	*<0.001*	***
Ant X 2.0 µg	-0.05	0.19	836.00	-0.24	0.809	
Early X GAD	0.07	0.19	836.00	0.36	0.722	
Late X GAD	-0.05	0.19	836.00	-0.29	0.776	
Peak X GAD	-0.13	0.19	836.00	-0.69	0.493	
Ant X GAD	-0.20	0.19	836.00	-1.08	0.280	
Dose 0.5 µg X GAD	0.22	0.19	836.00	1.18	0.237	
Dose 2.0 µg X GAD	0.14	0.19	836.00	0.72	0.471	
GAD X Age	0.00	0.01	836.00	0.12	0.904	
GAD X BMI	0.00	0.01	836.00	-0.30	0.763	
Early X 0.5 µg X GAD	*-0.58*	*0.27*	*836.00*	*-2.16*	*0.031*	*
Late X 0.5 µg X GAD	-0.07	0.27	836.00	-0.25	0.799	
Peak X 0.5 µg X GAD	-0.40	0.27	836.00	-1.52	0.129	
Ant X 0.5 µg X GAD	-0.04	0.27	836.00	-0.16	0.875	
Early X 2.0 µg X GAD	-0.13	0.27	836.00	-0.48	0.633	
Late X 2.0 µg X GAD	-0.10	0.27	836.00	-0.37	0.710	
Peak X 2.0 µg X GAD	-0.46	0.27	836.00	-1.74	0.083	^
Ant X 2.0 µg X GAD	0.02	0.27	836.00	0.06	0.954	

*Note: Sig. = significance. Coded as *** = p<0.001, ** = p<0.01, * = p<0.05, ^ = p<0.1*

© 2022 Teed AR et al. *JAMA Psychiatry*.

eTable 10. LME for BOLD PSC for the Meta-analysis–derived Self-processing vmPFC ROI.

Factor	Value	Std. Err.	DF	t-stat	p-value	Sig.
(Intercept)	0.00	0.11	836.00	0.01	0.990	
Early	0.02	0.07	836.00	0.25	0.805	
Late	-0.04	0.07	836.00	-0.52	0.606	
Peak	0.00	0.07	836.00	0.00	0.998	
Ant	*-0.16*	*0.07*	*836.00*	*-2.36*	*0.018*	*
Dose 0.5 µg	0.07	0.07	836.00	0.98	0.330	
Dose 2.0 µg	*0.23*	*0.07*	*836.00*	*3.45*	*0.001*	***
Group GAD	-0.02	0.14	836.00	-0.14	0.893	
Age	0.00	0.00	836.00	0.58	0.564	
BMI	0.00	0.00	836.00	-0.26	0.796	
Early X 0.5 µg	-0.07	0.10	836.00	-0.72	0.473	
Late X 0.5 µg	0.01	0.10	836.00	0.09	0.927	
Peak X 0.5 µg	*-0.32*	*0.10*	*836.00*	*-3.32*	*0.001*	***
Ant X 0.5 µg	0.04	0.10	836.00	0.43	0.670	
Early X 2.0 µg	*-0.52*	*0.10*	*836.00*	*-5.41*	*<0.001*	***
Late X 2.0 µg	0.01	0.10	836.00	0.12	0.907	
Peak X 2.0 µg	*-0.62*	*0.10*	*836.00*	*-6.43*	*<0.001*	***
Ant X 2.0 µg	-0.11	0.10	836.00	-1.16	0.249	
Early X GAD	0.04	0.10	836.00	0.39	0.695	
Late X GAD	-0.01	0.10	836.00	-0.13	0.894	
Peak X GAD	-0.05	0.10	836.00	-0.53	0.600	
Ant X GAD	-0.12	0.10	836.00	-1.23	0.220	
0.5 µg X GAD	0.19	0.10	836.00	1.95	0.052	^
2.0 µg X GAD	0.13	0.10	836.00	1.36	0.173	
GAD X Age	0.00	0.00	836.00	-0.36	0.720	
GAD X BMI	0.00	0.01	836.00	0.51	0.608	
Early X 0.5 µg X GAD	*-0.38*	*0.14*	*836.00*	*-2.83*	*0.005*	**
Late X 0.5 µg X GAD	-0.11	0.14	836.00	-0.79	0.429	
Peak X 0.5 µg X GAD	*-0.29*	*0.14*	*836.00*	*-2.17*	*0.031*	*
Ant X 0.5 µg X GAD	-0.16	0.14	836.00	-1.20	0.231	
Early X 2.0 µg X GAD	*-0.32*	*0.14*	*836.00*	*-2.34*	*0.019*	*
Late X 2.0 µg X GAD	-0.09	0.14	836.00	-0.65	0.519	
Peak X 2.0 µg X GAD	-0.21	0.14	836.00	-1.52	0.128	
Ant X 2.0 µg X GAD	0.08	0.14	836.00	0.61	0.542	

*Note: Sig. = significance. Coded as *** = p<0.001, ** = p<0.01, * = p<0.05, ^ = p<0.1*

© 2022 Teed AR et al. *JAMA Psychiatry*.

eTable 11. LME for BOLD PSC for the Meta-analysis–derived Valence vmPFC ROI.

Factor	Value	Std. Err.	DF	t-stat	p-value	Sig.
(Intercept)	0.00	0.10	836.00	-0.01	0.990	
Early	-0.04	0.06	836.00	-0.63	0.529	
Late	-0.03	0.06	836.00	-0.58	0.563	
Peak	*-0.15*	*0.06*	*836.00*	*-2.42*	*0.016*	*
Ant	*-0.15*	*0.06*	*836.00*	*-2.52*	*0.012*	*
Dose 0.5 µg	0.06	0.06	836.00	1.02	0.308	
Dose 2.0 µg	*0.25*	*0.06*	*836.00*	*4.08*	*<0.001*	***
Group GAD	0.09	0.13	836.00	0.68	0.498	
Age	0.00	0.00	836.00	0.29	0.776	
BMI	0.00	0.00	836.00	0.42	0.676	
Early X 0.5 µg	-0.12	0.09	836.00	-1.38	0.168	
Late X 0.5 µg	-0.01	0.09	836.00	-0.17	0.869	
Peak X 0.5 µg	*-0.19*	*0.09*	*836.00*	*-2.21*	*0.027*	*
Ant X 0.5 µg	-0.01	0.09	836.00	-0.07	0.944	
Early X 2.0 µg	*-0.63*	*0.09*	*836.00*	*-7.44*	*<0.001*	***
Late X 2.0 µg	-0.02	0.09	836.00	-0.19	0.852	
Peak X 2.0 µg	*-0.50*	*0.09*	*836.00*	*-5.85*	*<0.001*	***
Ant X 2.0 µg	-0.07	0.09	836.00	-0.83	0.405	
Early X GAD	-0.05	0.09	836.00	-0.64	0.526	
Late X GAD	-0.06	0.09	836.00	-0.75	0.454	
Peak X GAD	-0.15	0.09	836.00	-1.73	0.083	^
Ant X GAD	*-0.17*	*0.09*	*836.00*	*-1.96*	*0.050*	*
0.5 µg X GAD	0.08	0.09	836.00	0.98	0.327	
2.0 µg X GAD	0.03	0.09	836.00	0.41	0.684	
GAD X Age	0.00	0.00	836.00	0.00	0.997	
GAD X BMI	0.00	0.00	836.00	-0.15	0.883	
Early X 0.5 µg X GAD	-0.22	0.12	836.00	-1.79	0.074	^
Late X 0.5 µg X GAD	-0.01	0.12	836.00	-0.09	0.931	
Peak X 0.5 µg X GAD	-0.14	0.12	836.00	-1.16	0.248	
Ant X 0.5 µg X GAD	-0.03	0.12	836.00	-0.25	0.807	
Early X 2.0 µg X GAD	-0.08	0.12	836.00	-0.68	0.498	
Late X 2.0 µg X GAD	-0.01	0.12	836.00	-0.07	0.943	
Peak X 2.0 µg X GAD	-0.14	0.12	836.00	-1.17	0.243	
Ant X 2.0 µg X GAD	0.09	0.12	836.00	0.77	0.439	

*Note: Sig. = significance. Coded as *** = p<0.001, ** = p<0.01, * = p<0.05, ^ = p<0.1*

© 2022 Teed AR et al. *JAMA Psychiatry*.

eTable 12. LMEs for Heart Rate and Dial Response FPC Scores.

Factor	Value	Std. Err.	DF	t-value	p-value	Sig.
**Heart Rate Functional Principal Component 1**						
(Intercept)	-0.50	0.13	64.46	-3.78	<0.001	
Group GAD	0.22	0.19	64.46	1.18	0.241	
Dose 0.5 µg	*0.24*	*0.06*	*112.00*	*4.07*	*<0.001*	***
Dose 2.0 µg	*0.85*	*0.06*	*112.00*	*14.14*	*<0.001*	***
0.5 µg X GAD	0.15	0.09	112.00	1.75	0.083	
2.0 µg X GAD	-0.01	0.09	112.00	-0.11	0.913	
**Heart Rate Functional Principal Component 2**						
(Intercept)	0.25	0.05	79.09	5.21	<0.001	
Group GAD	0.05	0.07	79.09	0.80	0.426	
Dose 0.5 µg	*-0.18*	*0.03*	*112.00*	*-5.36*	*<0.001*	***
Dose 2.0 µg	*-0.56*	*0.03*	*112.00*	*-16.50*	*<0.001*	***
0.5 µg X GAD	*-0.11*	*0.05*	*112.00*	*-2.28*	*0.025*	*
2.0 µg X GAD	-0.07	0.05	112.00	-1.39	0.166	
**Dial Rating Functional Principal Component 1**						
(Intercept)	-0.49	0.09	133.35	-5.58	<0.001	
Group GAD	-0.04	0.12	133.35	-0.28	0.777	
Dose 0.5 µg	0.10	0.10	112.00	1.02	0.311	
Dose 2.0 µg	*1.13*	*0.10*	*112.00*	*11.37*	*<0.001*	***
0.5 µg X GAD	*0.39*	*0.14*	*112.00*	*2.79*	*0.006*	**
2.0 µg X GAD	0.21	0.14	112.00	1.47	0.144	
**Dial Rating Functional Principal Component 2**						
(Intercept)	0.12	0.06	152.58	2.22	0.028	
Group GAD	-0.01	0.08	152.58	-0.12	0.906	
Dose 0.5 µg	-0.11	0.07	112.00	-1.62	0.109	
Dose 2.0 µg	*-0.15*	*0.07*	*112.00*	*-2.23*	*0.028*	*
0.5 µg X GAD	-0.04	0.10	112.00	-0.41	0.680	
2.0 µg X GAD	-0.14	0.10	112.00	-1.44	0.152	

*Note: Sig. = significance. Coded as *** = p<0.001, ** = p<0.01, * = p<0.05, ^ = p<0.1*

© 2022 Teed AR et al. *JAMA Psychiatry*.

eTable 13. Multilevel Correlations between vmPFC Activity, Cardiac, and Subjective Responses During 0.5*μg* Isoproterenol Dose.

Parameter1	Parameter2	r-coeff	95% CI	t(56)	p-adj	p-sig
vmPFC_Peak	*HR*	*-0.51*	*[-0.68, -0.29]*	*-4.47*	*0.001*	**
Self.fpc1	*HR*	*0.52*	*[0.31, 0.69]*	*4.6*	*<0.001*	***
Val.fpc1	*HR*	*0.52*	*[0.31, 0.69]*	*4.59*	*<0.001*	***
Auto.fpc1	*HR*	*0.62*	*[0.43, 0.76]*	*5.87*	*<0.001*	***
vmPFC_Peak	*HR.fpc2*	*0.43*	*[0.19, 0.62]*	*3.52*	*0.020*	*
Self.fpc1	*HR.fpc2*	*-0.53*	*[-0.69, -0.31]*	*-4.63*	*<0.001*	***
Val.fpc1	*HR.fpc2*	*-0.47*	*[-0.65, -0.24]*	*-3.95*	*0.006*	**
Auto.fpc1	*HR.fpc2*	*-0.49*	*[-0.67, -0.27]*	*-4.24*	*0.003*	**
vmPFC_Peak	*RI*	*-0.44*	*[-0.63, -0.21]*	*-3.68*	*0.013*	*
Self.fpc1	*RI*	*0.49*	*[0.26, 0.66]*	*4.18*	*0.003*	**
Val.fpc1	*RI*	*0.41*	*[0.17, 0.61]*	*3.4*	*0.027*	*
Auto.fpc1	*RI*	*0.45*	*[0.21, 0.63]*	*3.74*	*0.011*	*
vmPFC_Peak	*HBI*	*-0.5*	*[-0.67, -0.28]*	*-4.34*	*0.002*	**
Self.fpc1	*HBI*	*0.54*	*[0.33, 0.70]*	*4.78*	*<0.001*	***
Val.fpc1	*HBI*	*0.44*	*[0.20, 0.63]*	*3.66*	*0.014*	*
Auto.fpc1	*HBI*	*0.44*	*[0.21, 0.63]*	*3.67*	*0.014*	*
vmPFC_Peak	CD25	0.14	[-0.12, 0.39]	1.09	0.719	
Self.fpc1	CD25	-0.08	[-0.33, 0.18]	-0.59	0.719	
Val.fpc1	CD25	-0.05	[-0.30, 0.21]	-0.36	0.719	
Auto.fpc1	CD25	-0.21	[-0.44, 0.05]	-1.59	0.706	
vmPFC_Peak	Dial	-0.38	[-0.58, -0.14]	-3.1	0.051	^
Self.fpc1	Dial	0.37	[0.12, 0.57]	2.94	0.071	^
Val.fpc1	Dial	0.29	[0.03, 0.51]	2.23	0.265	
Auto.fpc1	Dial	0.3	[0.05, 0.52]	2.36	0.218	
vmPFC_Peak	Anxious	-0.28	[-0.50, -0.02]	-2.18	0.267	
Self.fpc1	Anxious	0.33	[0.08, 0.54]	2.64	0.129	
Val.fpc1	Anxious	0.24	[-0.02, 0.47]	1.87	0.412	
Auto.fpc1	Anxious	0.32	[0.07, 0.53]	2.51	0.165	

*P-values adjusted for multiple comparisons via Hommel (1988), while confidence intervals (CI) are shown uncorrected. Significance coding: *** = p<0.001, ** = p<0.01, * = p<0.05, ^ = p<0.1. Abbreviations: vmPFC = ventromedial prefrontal cortex, Peak = peak effect of isoproterenol, Val = valence, Auto = autonomic, FPC = functional principal component, HR = heart rate, RI = respiratory intensity, HBI = heartbeat intensity, CD25 = chronotropic dose 25.*

eDiscussion 1. Study Limitations

Our focus on females with GAD was based on the fact that females outnumber males on a 2:1 basis[23]. Future research will need to establish whether the current results may extend to males. Recent studies have suggested a divergence of symptom clusters in GAD females vs. males such that females are more likely to report somatic symptoms whereas males are more likely to report mood and motor symptoms[24]. Thus, it is possible that the findings observed in the current study may represent a subtype expression of the disorder specific to females. Although we allowed participants who were taking psychotropic medications to enter the study, we do not think this impacted our findings, for several reasons: 1) they were stably medicated, 2) we would have expected to see disrupted action across all doses and epochs, and 3) the main effects of hypersensitivity and blunted vmPFC signal remained even after removing the medicated participants in follow-up analyses. Finally, given the high degree of comorbidity in GAD and our diagnostic focus, future work will need to test this paradigm in dimensionally and categorically defined samples (as in [25]) to see if a similar pattern of vmPFC hypoactivity during adrenergic stimulation is evident across other anxiety disorders.

© 2022 Teed AR et al. *JAMA Psychiatry.*

eReferences

1. Hassanpour MS, Simmons WK, Feinstein JS, Luo Q, Lapidus RC, Bodurka J, Paulus MP, Khalsa SS. The Insular Cortex Dynamically Maps Changes in Cardiorespiratory Interoception. *Neuropsychopharmacology.* 2018;43(2):426-434.
2. Hassanpour MS, Yan L, Wang DJ, Lapidus RC, Arevian AC, Simmons WK, Feusner JD, Khalsa SS. How the heart speaks to the brain: neural activity during cardiorespiratory interoceptive stimulation. *Philos Trans R Soc Lond B Biol Sci.* 2016;371(1708).
3. Khalsa SS, Feinstein JS, Li W, Feusner JD, Adolphs R, Hurlemann R. Panic Anxiety in Humans with Bilateral Amygdala Lesions: Pharmacological Induction via Cardiorespiratory Interoceptive Pathways. *J Neurosci.* 2016;36(12):3559-3566.
4. Khalsa SS, Rudrauf D, Sandesara C, Olshansky B, Tranel D. Bolus isoproterenol infusions provide a reliable method for assessing interoceptive awareness. *Int J Psychophysiol.* 2009;72(1):34-45.
5. Bates D, Maechler M, Bolker B, Walker S. Fitting Linear Mixed-Effects Models Using lme4. *Journal of Statistical Software.* 2015;67(1):1-48.
6. Kuznetsova A, Brockhoff PB, Christensen RHB. lmerTest Package: Tests in Linear Mixed Effects Models. *Journal of Statistical Software.* 2017;82(13):1-26.
7. Khalsa SS, Rudrauf D, Feinstein JS, Tranel D. The pathways of interoceptive awareness. *Nat Neurosci.* 2009;12(12):1494-1496.
8. Mills PJ, Dimsdale JE, Ancoli-Israel S, Clausen J, Loredo JS. The effects of hypoxia and sleep apnea on isoproterenol sensitivity. *Sleep.* 1998;21(7):731-735.
9. Makowski D, Been-Shachar MS, Patil I, Lüdecke D. Methods an Algorithms for Correlation Analysis in R. *Journal of Open Source Software.* 2019;5(51):2306.
10. Hommel G. A stagewise rejective multiple test procedure based on a modified Bonferroni test. *Biometrika.* 1988;75(2):383-386.
11. Pruessmann KP, Weiger M, Scheidegger MB, Boesiger P. SENSE: sensitivity encoding for fast MRI. *Magn Reson Med.* 1999;42(5):952-962.
12. Cox RW, Chen G, Glen DR, Reynolds RC, Taylor PA. FMRI Clustering in AFNI: False-Positive Rates Redux. *Brain Connect.* 2017;7(3):152-171.
13. Flournoy JC, Vijayakumar N, Cheng TW, Cosme D, Flannery JE, Pfeifer JH. Improving practices and inferences in developmental cognitive neuroscience. *Dev Cogn Neurosci.* 2020;45:100807.
14. Jiang L, Elrod C, Kim JJ, Swafford AD, Knight R, Thompson WK. Multi-Block Sparse Functional Principal Components Analysis for Longitudinal Microbiome Multi-Omics Data. 2021.
15. Lindquist KA, Satpute AB, Wager TD, Weber J, Barrett LF. The Brain Basis of Positive and Negative Affect: Evidence from a Meta-Analysis of the Human Neuroimaging Literature. *Cereb Cortex.* 2016;26(5):1910-1922.
16. Beissner F, Meissner K, Bar KJ, Napadow V. The autonomic brain: an activation likelihood estimation meta-analysis for central processing of autonomic function. *J Neurosci.* 2013;33(25):10503-10511.
17. Murray RJ, Schaer M, Debbane M. Degrees of separation: a quantitative neuroimaging meta-analysis investigating self-specificity and shared neural activation between self- and other-reflection. *Neurosci Biobehav Rev.* 2012;36(3):1043-1059.
18. Zhou D, Siegle G, Thompson W. A Bayesian model of activation and functional connectivity for event-related fMRI. n.d.:1-3.
19. Venables WN, Ripley BD. *Modern Applied Statistics with S*. Fourth Edition ed. New York: Springer; 2002.
20. Bates DG, Maechler M. Matrix: Sparse and Dense Matrix Classes and Methods. R Package. Retrieved from https://cran.r-project.org/package=Matrix.
21. Borchers HW. pracma: Practical Numerical Math Functions. R package. Retrieved from https://cran.r-project.org/package=pracma.
22. Team RC. R: A language and environment for statistical computing. . Foundation for Statistical Computing. Available at: Retrieved from https://www.r-project.org/.
23. Craske MG. *Origins of phobias and anxiety disorders. Why more women than men?*: Elsevier; 2003.
24. Thompson KN, Hubel C, Cheesman R, Adey BN, Armour C, Davies MR, Hotopf M, Jones IR, Kalsi G, McIntosh AM, Monssen D, Peel AJ, Rogers HC, Skelton M, Smith DJ, Walters JTR, Breen G, Eley TC. Age and sex-related variability in the presentation of generalized anxiety and depression symptoms. *Depress Anxiety*. 2021;38(10):1054-1065.

© 2022 Teed AR et al. *JAMA Psychiatry.*

25. Marin MF, Hammoud MZ, Klumpp H, Simon NM, Milad MR. Multimodal Categorical and Dimensional Approaches to Understanding Threat Conditioning and Its Extinction in Individuals With Anxiety Disorders. *JAMA Psychiatry*. 2020;77(6):618-627.

© 2022 Teed AR et al. *JAMA Psychiatry*.
