# Supplementary material for: Association of Generalized Anxiety Disorder With Autonomic Hypersensitivity and Blunted Ventromedial Prefrontal Cortex Activity During Peripheral Adrenergic Stimulation: A Randomized Clinical Trial
Source: JAMA Psychiatry. 2022 Feb 2;79(4):323–32. doi: 10.1001/jamapsychiatry.2021.4225 (PMC8811711; doi:10.1001/jamapsychiatry.2021.4225)
Supplement: Supplement 2. — Trial Protocol [file jamapsychiatry-e214225-s002.pdf]

Title of Project: Neural basis of interoceptive dysfunction and anxiety in anorexia nervosa

Principal Investigator: Sahib Khalsa, M.D., Ph.D.

Address:
Laureate Institute for Brain Research
6655 S Yale Ave
Tulsa, OK 74136-3326

Phone:
918-502-5743

Fax:
918-502-5135

Email:
skhalsa@laureateinstitute.org

Sponsors:
Laureate Institute for Brain Research
http://www.laureateinstitute.org

A. Purpose of the study

In order to sustain life, the human brain must decipher a multitude of complex signals coming from outside and inside of the body and maintain a healthy balance. Several psychiatric and cardiovascular disorders manifest through distorted sensitivity to internal body signals like the heartbeat and the breath, suggesting that the neural processes responsible for decomposing these signals may be dysfunctional. Much of the previous research on these sensations has been limited by methodological weaknesses, including the lack of appropriate techniques, small sample sizes, no longitudinal assessments, and failure to examine causal neural mechanisms. We have previously addressed some these weaknesses through the development of highly specific pharmacological probes of cardiac and respiratory sensation. In the current proposal, we propose to utilize one of these probes, isoproterenol, a rapidly metabolized peripheral beta adrenergic agonist similar to epinephrine (adrenaline), to further examine visceral sensation in healthy individuals, and separate groups of patients with eating disorders, anxiety disorders, depression, and focal brain injury during functional neuroimaging. This series of studies therefore aims to conclusively locate the brain regions responsible for encoding cardiorespiratory sensations and determine whether they are dysfunctional in individuals affected by anxiety, depression, and eating disorders. We further aim to explore whether an intervention involving breathing modulation attenuates anxiety, reduces interoceptive symptom burden and improves outcomes in individuals with cardiac arrhythmias. By evaluating the same interoceptive sensations across different human illnesses, we hope to provide convergent evidence resulting in identification of core underlying neural processes, and to discern relative contributions in each condition. By testing a novel intervention we hope to provide initial links to therapeutic treatments for interoceptive dysfunction. We will improve upon prior investigations by using larger sample sizes, longitudinal follow up, and examining causal and compensatory neural mechanisms by integrating the human lesion method with functional brain imaging. This systematic program of research has the potential to conclusively define the roles of cardiorespiratory interoceptive sensation in these selected psychiatric and cardiac populations, identify the underlying psychophysiological and neural correlates, and could lead to new treatments.

Specific Aim1: Identify the brain regions responding to cardiorespiratory interoceptive sensations during bolus isoproterenol and saline infusions in healthy individuals.

Hypothesis 1: Bolus isoproterenol infusions will increase cardiorespiratory sensations and induce dose-related increases in activation of previously implicated brain regions including the insula, anterior cingulate and somatosensory cortex in healthy individuals.

Specific Aim 2: Evaluate the effects of bolus isoproterenol and saline infusions on brain activity in individuals with anorexia nervosa (AN), panic disorder (PD), Generalized Anxiety Disorder (GAD) and major depressive disorder (MDD).

Hypothesis 2a: AN, PD and GAD individuals will display abnormally increased activation of the insula, anterior cingulate and somatosensory cortices relative to healthy comparisons (HC) during saline and isoproterenol infusions. MDD individuals will display abnormally decreased activation of these same regions relative to HCs during saline and isoproterenol infusions. These neural responses will be associated with clinical symptoms and will longitudinally predict illness trajectory over 1 year.

Hypothesis 2b: AN and PD be differentiated from GAD by increased activation in

interoceptive brain regions (insula, anterior cingulate and somatosensory cortices), while GAD and MDD will display increased activation of brain regions associated with rumination and worry (dorsolateral prefrontal cortex) relative to the other two conditions.

B. Background and Significance

The human brain has constant access to a multitude of complex signals, which it must simplify and organize in order to sustain the integrity of the organism. Many of these signals originate from outside of the body, such as lights, sounds, and smells, and much is known about how humans consciously perceive these exteroceptive signals and how the human brain represents them. Comparatively little is known about how the human brain processes interoceptive signals originating from inside of the body, despite the fact that the brain has access to far more of them (for instance, intestinal tension, bladder distension, breath, heartbeat, body temperature, blood pressure, serum osmolality, serum pH, inflammation, proprioception etc.).

Knowing how our brains encode these interoceptive feelings is as vital for ensuring survival as the ability to see and hear external threats, as any individual with diabetes or myocardial infarction survivor can tell you. Several psychiatric disorders are even diagnostically characterized by disrupted interoception: for instance, individuals with panic disorder (PD) experience prominent interoceptive sensations during panic attacks including palpitations, difficulty breathing and dizziness. They report hypersensitivity these signals, and these symptoms are core features of the defining criteria of panic attacks [1]. Similarly, individuals with generalized anxiety disorder (GAD) frequently complain of exaggerated physical symptoms, and worry excessively about their harmful health consequences despite the absence of corroborating evidence [2]. Individuals with eating disorders also frequently report exaggerated fullness in response to small meals or complain of bladder and bowel fullness outside of meal times [3]. They are also more likely to have anxiety disorders [4], raising the question of whether their interoceptive disturbances arise from the eating disorder, the anxiety disorder, or a process subsuming both as recently suggested by prominent eating disorders experts [5]. The overlapping interoceptive hypersensitivities across these disorders raises the possibility that they share a common underlying dysfunction in the interoceptive neural system, although this has never been systematically investigated.

While anxiety and eating disorders are characterized by interoceptive hypersensitivity, major depressive disorder (MDD) exhibits the opposite pattern. Beyond the low appetite, anhedonia and poor energy frequently exhibited by individuals with MDD, emerging studies are suggesting a correspondingly reduced awareness of cardiac sensations [6-8]. A recent study by Laureate Institute for Brain Research (LIBR) Principal Investigator Dr. W. Kyle Simmons (sub-investigator on this proposal) also suggests that reduced activation of the insular cortex, a prominent node within the interoceptive neural system, accompanies this disorder when directing attention to this interoceptive signal under resting conditions [9]. Unfortunately, these prior studies are limited in the approach to measuring cardiac awareness: they measure symptoms only under physiological resting conditions, when the majority of individuals never even report awareness of these sensations [10]. Furthermore, several of the tasks employed suffer from methodological limitations: the most commonly employed task, heartbeat tracking, fails to provide a statistical measure of perception at the individual level, is susceptible to bias based on prior knowledge of average heart rate, and is insensitive to actual changes in heart rate [11]. The isoproterenol infusion task overcomes all of these limitations by assessing biased beliefs about heart rates (via inclusion of saline placebo

conditions), by modulating heart rates (to elicit changes in sensation in all participants) and providing individual statistical measures (via cross correlation scores at each dose).

C. Preliminary Studies and Description of Laboratory:

Investigator background: The principal investigator of this protocol, Sahib Khalsa MD, PhD, is a board-certified psychiatrist who adapted the isoproterenol infusion protocol to measure IA in humans. He has successfully applied it in over 110 human participants (healthy subjects, psychiatric patients with AN, PD, GAD, brain injury patients with herpes simplex encephalitis causing insula damage, and UWD patients with amygdala damage) without any adverse events, across two institutions (University of Iowa and UCLA). Dr. Khalsa has a robust publication record demonstrating the adaptation of the isoproterenol method for studying interoceptive awareness and its application within psychiatric and brain injury patient samples, in premier neuroscience and psychiatric journals. Of the additional investigators on the protocol (Justin Feinstein PhD, Kyle Simmons PhD, Jerzy Bodurka PhD, and Martin Paulus MD), one is a board-certified licensed psychiatrist (Martin Paulus MD), and another is a clinical psychologist (Justin Feinstein PhD). Both can assist the principal investigator in the event that any adverse psychological effects should arise during the course of the study.

Rationale for isoproterenol infusion approach: Most prior studies attempting to study the conscious perception of interoceptive sensations (a process called interoceptive awareness or IA), have used non-invasive approaches. While these studies may carry less risk and are easier to conduct, they suffer from many limitations. With respect to measuring cardiovascular sensation, the most commonly employed tasks have been heartbeat perception tasks. In these tasks, subjects attend to their heartbeat sensations and then report whether their subjective perception matches with the objectively measured heartbeat parameter. Important insights into the limited validity of these tasks have been gleaned across 40 years of studies using these tasks. For instance, these tasks frequently carry a high degree of task difficulty. They can be fatiguing, and many lack of statistically valid dependent measures of perceptual accuracy [10, 12-15]. Furthermore, these tasks have typically assessed IA during physiological rest, a state in which the majority of participants are unable to detect their heartbeat [13]. Even more concerning is that some of these tasks have been shown to be insensitive to changes in arousal [16], calling into question their face validity. These limitations led us to previously develop the isoproterenol approach proposed for the current set of studies. Isoproterenol is a fast acting, peripheral beta adrenergic agonist similar to epinephrine (adrenaline) that elicits dose dependent increased in heart rate and contractility [17]. These pharmacological effects are extremely brief in duration due to its very short half-life and rapid metabolism. We have previously shown that bolus intravenous isoproterenol infusions produce reliable, dose dependent, brief changes in cardiovascular arousal (<2 minutes). These changes in arousal are accompanied by dose dependent increases in IA, which we index by asking participants to rotate a dial in real time during each infusion, to report their moment-to-moment awareness of the intensity of these sensations (figure 1) [10]. The isoproterenol infusions approach has been safely applied in hundreds of studies with human participants across more than a 40 year period [17-20], and those studies directly informed the development of the currently proposed protocol.

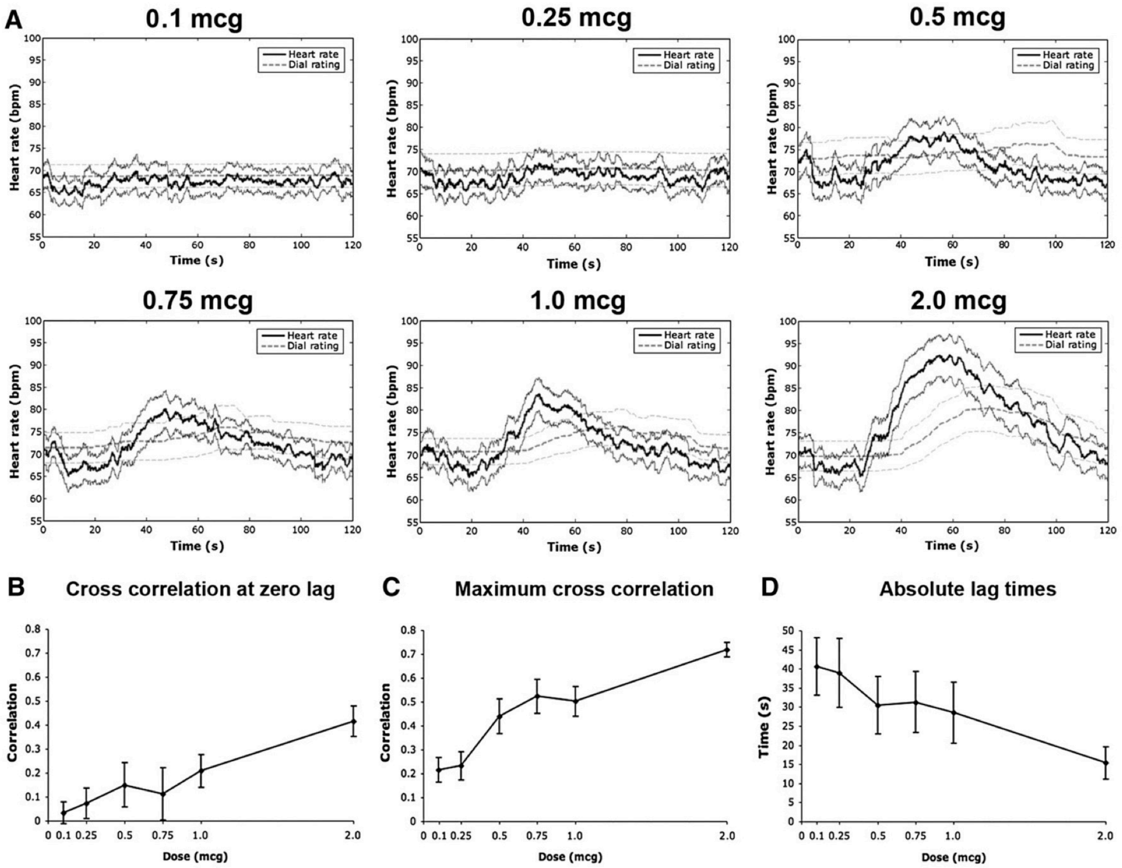

Figure 1. Time course of heart rate and interoceptive dial ratings during bolus isoproterenol infusions. (A) Mean time course of heart rate (thick black line) and associated dial rating (thick dashed gray line). Thin lines indicate SE mean. (B) Cross correlations between heart rate increases and subjective ratings of the heartbeat and breathing intensity at zero lag. (C) Maximum cross correlation. (D) Absolute lag times vary as a function of the maximum cross correlation.

Laboratory description: The experimental setup (figure 2), involves the intravenous delivery of bolus infusions of isoproterenol and saline. Infusions are delivered in a double blinded manner in order to control for the effects of expectancy on subjective ratings. All participants are always informed they will receive isoproterenol and saline during the experiment, but that neither they nor the experimenter will know the identity of any particular infusion. During the infusions all participants' psychophysiological parameters are always continuously monitored during the infusions (lead 2 ECG to provide heart rhythm and heart rate, respiratory rate, skin conductance), and vital signs are always measured before and after each infusion session. All participants are pre screened with a 12 lead EKG, urine pregnancy (when applicable) and drug screening, vital sign measurement and a careful medical history (including medication use, family history of cardiovascular disease) is taken and a physical exam is performed to evaluate for the presence of exclusionary conditions. On the day of testing, several of these measures are again repeated (urine pregnancy, urine drug screening, vital sign measurement).

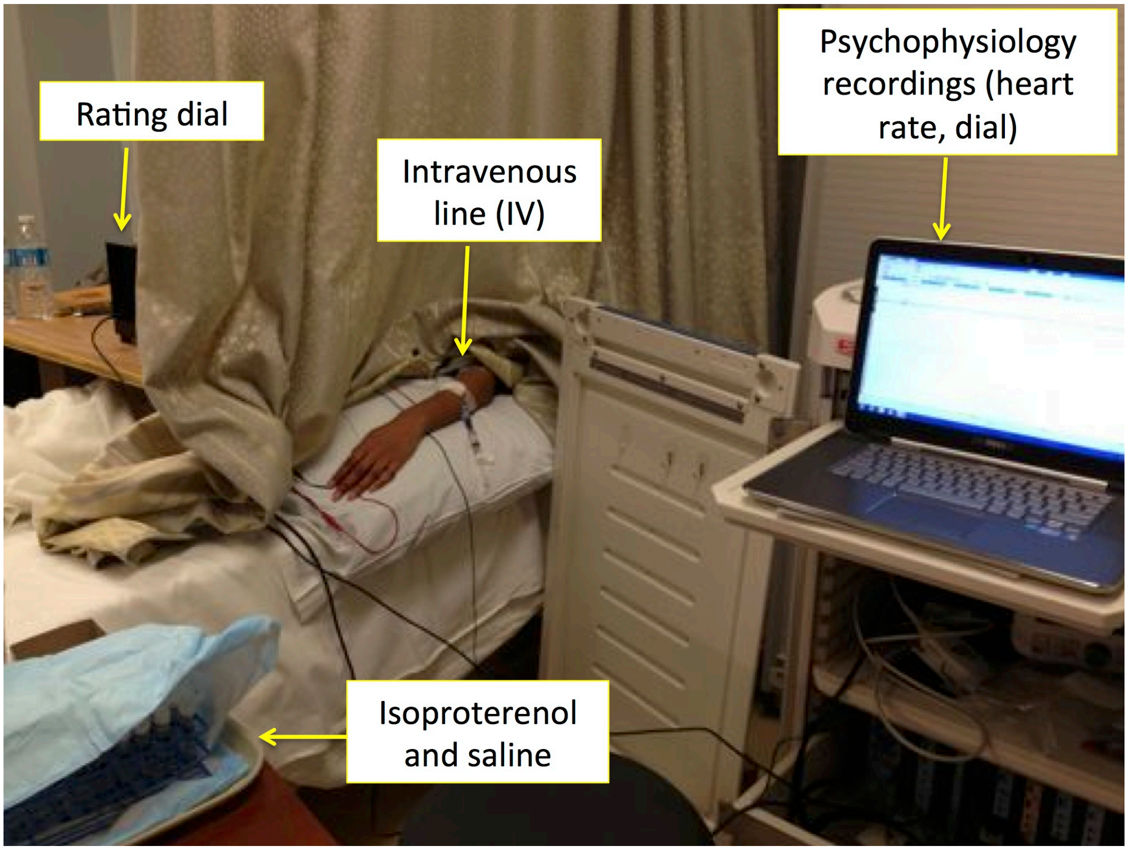

Figure 2. Experimental setup (non-MRI setting). Participants are hidden from the experimenter's view (and vice versa) using a curtain allowing access to the intravenous line. Infusions are delivered only by an experienced clinician present in the room (MD or RN). Participants remain in verbal communication with the experimenters at any time before, during or after infusion delivery. Saline and isoproterenol are packaged by a registered pharmacy into identical 3cc syringes. During the infusions, subjects rotate a dial placed on a table in front of them, to indicate their ongoing experience of the intensity of heartbeat and breathing sensations. All data are recorded on a computer for data analysis.

<u>Preliminary studies (functional neuroimaging)</u>: In order to identify brain regions responding to changes in cardiovascular arousal, we have begun measuring brain activation occurring in response to bolus infusions of isoproterenol. Participants underwent functional magnetic resonance imagine (fMRI) while receiving bolus isoproterenol and saline infusions (figure 3). We have been using a multimodal neuroimaging approach, combining conventional Blood Oxygen Level Dependent (BOLD) imaging with Arterial Spin Labelling (ASL), to differentiate potential effects of the medication on arterial blood flow from neural activation. This approach has been previously approved by the principal investigators former institutional IRB (UCLA), resulting in successful data collection from 23 healthy participants inside the MRI scanner without any adverse events. Here, we propose to continue this approach by conducting isoproterenol infusions studies in the fMRI environment.

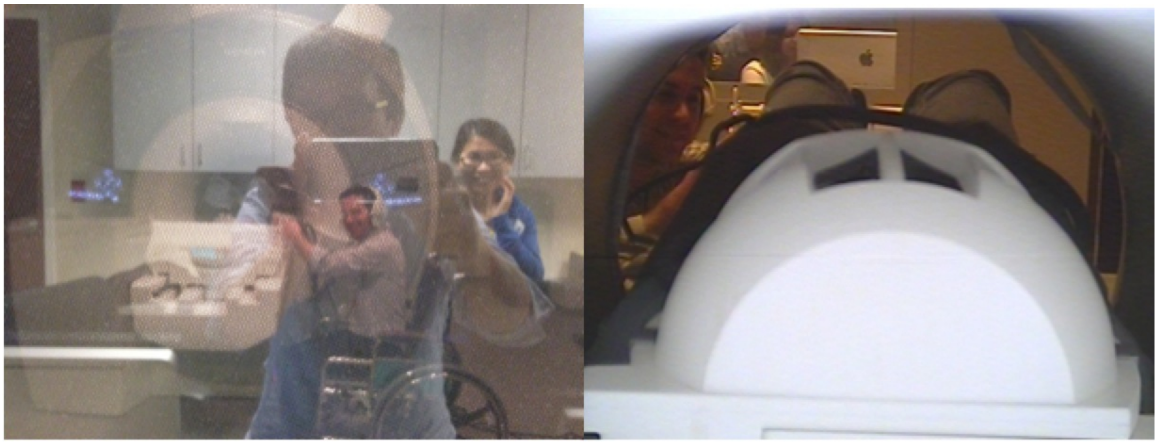

Figure 3. Experimental setup (MRI setting). Infusions are delivered while participants are inside of the scanner, only by an experienced clinician (MD or RN; left panel) present in the scanner room. Participants remain in verbal communication with the experimenters at any time before, during or after infusion delivery. During the experiment two members of the research team operate the MRI scanner and monitor the physiological parameters from the control room (left and right panels).

Preliminary studies (psychiatric disorders): A study completed at the former institution (UCLA) evaluated IA using the isoproterenol method in a group of 17 individuals with AN and 17 healthy comparisons [21]. We found that individuals with AN demonstrated abnormal IA, particularly when anticipating eating a meal. Here, we propose to continue this approach by conducting isoproterenol infusions studies in the fMRI environment, and to extend this to two additional patient populations (AN, PD, GAD and MDD). We have previously successfully applied this approach with psychiatric individuals in the fMRI environment in 3 individuals with AN, and 2 individuals with PD (figure 4).

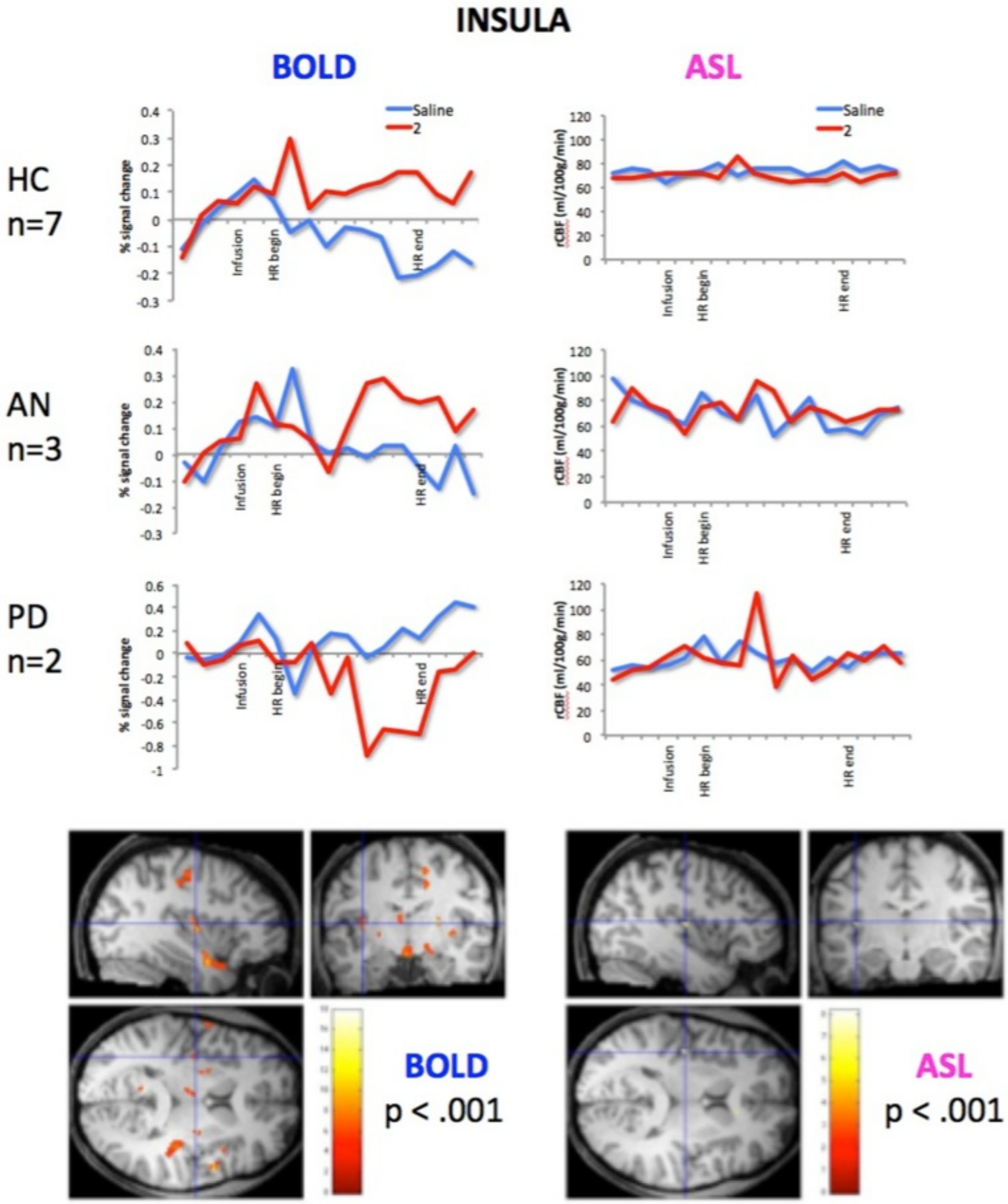

Figure 4. Top. Mean BOLD & ASL time series for the insula in 7 healthy comparisons (HC), 3 anorexia nervosa (AN), and 2 panic disorder (PD) participants. Bottom: Voxelwise whole brain analysis for the HC (uncorr). Data were collected under IRB authorized protocol at the previous institution (UCLA).

D. Research Design and Methods:

Overall protocol design

Pharmacological intervention

a. Participants will rate their experience of body sensations and emotions during and after receipt of isoproterenol and saline infusions, while outside and inside of the MRI scanner at LIBR. Some participants may receive a topical anesthetic skin cream while undergoing isoproterenol and saline infusions.
b. Participants enrolled: AN, PD, GAD, brain injury and healthy participants
c. Dose exposure: bolus doses of isoproterenol from 0.1 to 4.0 mcg. Maximum daily dose exposure will not exceed 25 mcg (consistent with dose exposure from Khalsa et al, 2015). Topical anesthetic dose will be 4% lidocaine, not to exceed 60g (two 30g tubes).
d. Duration: up to 5 visits, 3 on site and 2 remote follow up visits.

Once a participant successfully meets the phone screening inclusion and exclusion criteria they will be assigned to one of two arms: 1) pharmacological intervention or 2) breathing intervention. After providing their informed consent they will participate in a face-to-face medical screening visit to verify eligibility for the intervention specific to their assignment.

Participants in the pharmacological intervention may be asked to complete a number of different self-report, physiological, and behavioral measures outside and inside of the MRI scanner (described below in ‘psychophysiological assessments’).

Recruitment

We aim to recruit a total of 410 human participants over 5 years (see ‘Human Participants’ section for details). Participants will be recruited through a pre-approved LIBR Screening protocol (WIRB # 20101611), through the LIBR database, through flyers and social media advertisements, and through word of mouth via cardiologists in the community.

Screening assessment:

Participants will be required to complete a phone screening assessment prior to study entry. Upon clearing this assessment they will be consented into the study. Before completing any psychophysiological or neuroimaging assessments they will also be required to complete a face to face evaluation of their psychiatric and medical condition.

Self report assessments:

Two forms of self report may be collected for ancillary analyses of brain imaging data: demographic/medical questionnaires, and measures of psychiatric health. The demographic/medical questionnaires assess broad demographic information, as well as medical history and current health. Measures of psychiatric health will include common screeners for psychiatric illness, state and trait measures of physical, mental and social wellbeing, and psychiatric symptom severity (see Assessment List).

Psychiatric and Medical Evaluations:

All participants consenting to study entry will be asked a series of standardized questions about psychiatric or medical symptoms they have experienced during their lifetime, including a detailed medication history and family history. An unstructured psychiatric interview and a Structured Clinical Interview for DSM-IV Disorders (SCID) or the M.I.N.I. may be administered as part of the determination of eligibility. These assessments will be obtained through the LIBR Screening protocol. All subjects in the pharmacological intervention will undergo a physical exam including measurement of vital signs, recording of a 12-lead EKG to be evaluated by a board cardiologist, submission of urine sample for pregnancy and drug screening. During this visit many of the self report assessments may be completed.

Psychophysiological assessments:

A) Pharmacological intervention (outside of MRI scanner)

Pharmacological intervention

<u>Heartbeat Tapping</u>: In this task, which provides a different measure of interoceptive accuracy, participants may be asked to tap a keypad each time they feel their heartbeat during several different trials.

Trial 1 – Heartbeat tracking (60s): “Please tap this device in synchrony with your heartbeat. Even if you cannot feel your heartbeat, try to mirror it as closely as possible by tapping every time your heart beats.” Trial 1 provides a low threshold measure of an individual’s belief about their resting heart rate.

Trial 2 – Tone tracking (60s): “You will hear a series of tones played in your headphones. Please tap this device in synchrony with the tones. Each time you hear a tone, try to mirror it as closely as possible by tapping on this device.” Trial 2 is used as an exteroceptive control condition.

Trial 3 – Heartbeat awareness (60s): “It can be very difficult to feel your heart beating at rest. For this trial, only tap the device when you actually feel your heart beat. If you do not feel your heart beat, then you should not tap the device. We have found that some people go the whole trial without tapping at all. Please try your best and only tap the device during the moments when you actually feel your heart beat.” Trial 3 provides a high threshold measure for an individual’s awareness of their heartbeat sensations.

Trial 4 – Heartbeat awareness with inspiratory breath hold (60s): “This trial will be the same as the previous trial. Only tap the device when you actually feel your heart beat. If you do not feel your heart beat, then you should not tap the device. This time, however, we would like you to empty your lungs of all the air, and then take as deep of a breath in as possible and hold it for as long as you can tolerate. While you’re holding your breath, please try your best and only tap the device during the moments when you actually feel your heart beat.” Trial 4 examines the degree to which accuracy of heartbeat awareness improves following an interoceptive disturbance.

Breath Hold Challenge: This task will have participants undergo 2 inspiratory breath holds, providing a brief measure of interoceptive distress tolerance and carbon dioxide sensitivity [22]. The maximum trial length is 2 minutes, and there will be at least a 2-minute rest between trials. Participants are instructed to hold their breath for as long as they can tolerate following a vital capacity inhalation. The duration of each breath hold will be calculated starting from the moment when they finish inhaling and ending the moment they exhale. All participants will need to wear a nose clip to ensure they are not exhaling any air during the breath hold.

Cold Pressor Challenge: This task, which is a common laboratory measure of interoceptive pain tolerance, has participants immerse their hand in a circulating pool of water cooled to between 0-6 degrees Celsius. Participants will be asked to keep their hand in the water for as long as they can tolerate, providing a brief measure of pain/stress tolerance and emotional reactivity/regulation. During each immersion participants will provide real-time ratings of their degree of pain using an electronic visual analog scale ranging from no pain at all to the worst pain imaginable. The Cold Pressor paradigm is a gold standard paradigm that has been repeatedly used over the past century to safely induce transient states of intense pain [23, 24]. Maximum trial length is 5 minutes.

Isoproterenol and Saline Challenge: this task assesses multiple aspects of interoceptive awareness, including detection thresholds, symptom magnitudes, discrimination (relative to saline), and interoceptive accuracy. Participants will rate their experience of physical sensations and emotions before, during and after receiving bolus isoproterenol and saline infusions. These infusions may be delivered inside and outside of the MRI scanner. All participants receiving infusions inside of the scanner will first receive infusions outside of the scanner. This ensures that each participant is familiar with the sensations elicited by isoproterenol prior to entering the scanning environment. It is also used to establish the chronotropic dose 25 (CD25), or the isoproterenol dose necessary to increase the participant's heart rate by 25 beats per minute above baseline. The CD25 is a commonly reported measure of beta adrenergic receptor sensitivity [20, 25] that is important to measure as it has been found to vary in underweight AN [26]. Subjects will be notified via visual or auditory prompt of the onset of each infusion. They will be instructed to attend to their heartbeat and breathing sensations, and rate the intensity of these sensations by rotating a dial. Infusions are administered a minimum of 3 min apart. Heart rhythm and rate (via lead II ECG) will be continuously recorded and monitored throughout each infusion by a clinically trained member of the study team (RN or MD).

Meal Challenge: participants may be asked to consume a 300 Calorie snack upon study entry and a 1000 Calorie meal at the end the infusion session. These meals serve several purposes. They allow for the standardization of the gastric interoceptive system prior to study data collection. They allow for nourishment during prolonged testing conditions (e.g., experiments lasting > 5 hours). They enable select populations (e.g. AN) to maintain adherence to their meal plan. Finally, they also allow for examination of the influence of gastric interoceptive stimulation on cardiorespiratory parameters in select populations (e.g., AN). The 1000 Calorie meal is slightly larger than a typical outpatient meal plan, though identical to our prior study (Khalsa et al, 2015). While participants will be asked to complete the full meal, this will not be enforced. Therefore, participants not eating the full meal may continue study participation. Meal duration and calories consumed will be recorded.

B) Pharmacological intervention (inside of MRI scanner)

Heartbeat Attention: During MRI scanning participants may be asked to complete a task measuring neural correlates of interoceptive attention [27]. Participants will engage in two types of trials: the interoception condition and the exteroception condition. During the interoception condition, the word "HEART" or "STOMACH" is presented on the screen and subjects are instructed to focus their attention on interoceptive sensations from that organ. For example, upon seeing the word "HEART", participants focus on how intensely they can feel the sensation of their heart beating. During the exteroception control condition, the word "TARGET" is presented in the middle of the screen and the color of the word alternates from black to a lighter shade of gray every second. Participants are instructed to focus their attention on the intensity of these color changes. Each task condition is presented in 10-second blocks, and half of the blocks are followed immediately by a 5-second response period during which the participant uses a visual analog scale (1-to-7) to rate the intensity of interoceptive sensations or exteroceptive color changes experienced during the preceding trial. Each block is separated by a variable inter-stimulus interval, during which participants look at a fixation mark. Each run of the task begins with a 10-sec initial fixation period and ends with a 10-sec final fixation period. Participants will perform up to 3 scanning runs, each lasting 360 seconds (including initial and final fixation periods).

Isoproterenol and Saline Challenge: During MRI scanning participants will rate their experience of physical sensations and emotions before, during and after receiving bolus isoproterenol and saline infusions. All participants receiving infusions inside of the scanner will first receive infusions outside of the scanner, ensuring that each participant is familiar with the sensations elicited by isoproterenol prior to entering the scanning environment. Subjects will be notified via visual or auditory prompt of the onset of each infusion. They will be instructed to attend to heartbeat and breathing sensations, and rate the intensity of these sensations by rotating an MRI compatible dial, as with the behavioral protocol. They will be instructed to attend to their heartbeat and breathing sensations, and rate the intensity of these sensations by rotating a dial. Infusions are administered a minimum of 3 min apart. Heart rate will be continuously recorded (via lead II ECG or pulse oximeter) and monitored by a clinically trained member of the study team present in the room (RN or MD) throughout the entire infusion session duration.

Psychophysiological Procedures

Only participants meeting eligibility criteria who have successfully passed the screening visit will be asked to complete psychophysiological assessments. After verifying continued eligibility (recording vital signs, collection of urine drug/pregnancy screen and physical exam), they will receive IV placement by a trained RN staff member. Prior to completing the psychophysiological assessments, task instructions will be explained and participants will complete a question and answer session to ensure that they understand all of the tasks. After each set of tasks vital signs will be recorded to ensure continued study eligibility. Heart rhythm and rate (via lead II ECG) will be continuously recorded and monitored by a clinically trained member of the study team present in the room (RN or MD) throughout the entire infusion session duration.

MRI Procedures (pharmacological intervention only)

Only participants meeting eligibility criteria who have successfully passed the screening visit will complete the MRI assessments. After verifying continued eligibility (recording

vital signs, collection of urine drug/pregnancy screen and physical exam), participants will receive IV placement by a trained RN staff member.

Prior to scanning, task instructions will be explained and participants will perform a short pre-scanning task training session to ensure that they understand all of the fMRI tasks. They will also have the option to lie in LIBR's mock scanner in order to acclimate to the environment. All participants will be provided with earplugs and/or sound attenuating headphones as protection from scanner sounds.

Participants will be placed in the magnet with the investigator ensuring that the participant is both comfortable, able to fully see and hear the stimuli, able to comfortably manipulate any required response apparatus, and trained to use the scanners emergency call button. The emergency call button is a hand-held response apparatus that allows the participant to request the investigator/MR Technologist's immediate attention.

During scanning, bolus infusions will be delivered by a clinically trained member of the study team present in the room (RN or MD). During MRI scanning, heart rate and/or rhythm will be continuously recorded and monitored (via pulse oximeter or lead II ECG) by a clinically trained member of the study team present in the room (RN or MD) throughout the entire infusion session duration.

MRI scans will be acquired on a 3-tesla GE Discovery MR750 scanner (GE Medical Systems) and 32 channel phased-array coil system (Nova Medical). At the beginning of any scan session a series of 15-second MRI localizer scans will be obtained in order to prescribe locations of the subsequent anatomical and functional scans. Next, a series of high-resolution anatomical scans, used both to localize and align functional scans, will be obtained. These anatomical scans may also be used for analysis of anatomical features, such as cortical thickness and volumetric measurements. Before the anatomical scans, participants will be instructed as to the type of scan being performed and asked to remain as still as possible. High-resolution anatomical scans take 6 minutes to complete. Prior to each functional scan, participants will be reminded of the task instructions and the participant's comfort level will be assessed. A typical functional imaging session for this study will involve a series of five 7-10 minute scans with short breaks of approximately 1-minute between scans. After scanning, all participants will be debriefed regarding the objectives of the study. The participant's total amount of time spent in the scanner will never exceed 2 hours in any one session.

Physiological measurement during MRI scanning:

Physiological measures including heart rate, respiration rate, electroencephalographic (EEG) brain activity and electrocardiography (ECG) activity may be measured during functional fMRI scanning [28]. The scanner is equipped with a respiration belt that fits comfortably around the subject's chest to measure respiratory changes and a finger pulse oximeter for measuring heart rate and blood oxygenation. Electrodermal activity will be recorded using MRI-compatible electrodes placed on the volar surfaces of the hand or foot. Recordings will be sent to a BIOPAC Systems, Inc. data acquisition system located in the scanner control room (outside the MRI magnet room), via MRI-compatible leads that go through a patch panel dividing the MRI magnet and the scanner control room. Exhaled end-tidal CO_2 concentration will be recorded utilizing nasal cannula and plastic tubing connected to a standard transducer attached to the BIOPAC system. All physiological signals will be passively measured and no electrical

currents will be applied to the subject. EEG and ECG recordings will be sent to a dedicated data acquisition system via MRI-compatible optical cables that go through a patch panel dividing the scanner and control room.

During MRI scanning, heart rate and/or rhythm will be continuously monitored by a clinically trained member of the study team present in the room (RN or MD) throughout the entire infusion session duration. Participants displaying prolonged elevations in pulse rate (e.g. >140 bpm) will be asked via intercom to report their anxiety level, and indicate whether they wish to continue the experiment. Subjects reporting increased anxiety levels at this or any time during the scanning will be offered the opportunity to leave the scanner and discontinue the study. Any participant displaying abnormally high elevations in pulse rate (e.g. >160 bpm) will be excused from the scanner. A clinically trained member of the team (MD or RN) will always be inside of the scanner room administering the infusions and monitoring the heart rate. Each participants' vital signs will be measured immediately upon exiting the MRI room. In the event of emergency, the first responder alerting procedures will be followed, as specified in the emergency protocol section (below).

Physiological measurement during behavioral testing:

Heart rate (ECG), respiration, skin conductance, and pulse oximetry, will be recorded continuously using BIOPAC instrumentation (Goleta, California) during each of the behavioral tasks that were described above (with the exception of the Heartbeat Counting task which only utilizes heart rate). We will also collect end-tidal oxygen and carbon dioxide levels using an Oxigraf O2 Capnography system (Mountain View, California) before and after each Breath Hold Challenge. Other non-invasive physiological measures that may be employed include cardiac impedance (Biopac, Goleta, California) and continuous blood pressure (CNAP 500 monitor, CN Systems, Graz, Austria).

During psychophysiological assessment, heart rate and/or rhythm will be continuously monitored by a clinically trained member of the study team present in the room (RN or MD) throughout the entire infusion session duration. Participants displaying prolonged elevations in pulse rate (e.g. >140 bpm) will be asked to report their anxiety level, and indicate whether they wish to continue the experiment. Subjects reporting increased anxiety levels at this or any time during the assessment will be offered the opportunity to discontinue the study. Any participant displaying abnormally high elevations in pulse range (e.g. >160 bpm) will be excused from the study. A clinically trained member of the team (RN or MD) will always be inside of the room administering the infusions and monitoring the heart rate. Each participants' vital signs will be measured immediately upon finishing each infusion assessment. In the event of emergency, the first responder alerting procedures will be followed, as specified in the emergency protocol section (below).

E. Statistical Methods:

Data will be analyzed using standard univariate and multivariate statistical parametric methods except for instances that require the use of non-parametric statistical analysis. Examples of these methods include analysis of variance (ANOVA), t-tests, multi-dimensional scaling, correlation analyses, clustering algorithms and functional connectivity. Both within- and between-subject effects will be explored, and whenever possible, a repeated measures mixed-model ANOVA design will be utilized to examine

group by time interactions. MRI data will be analyzed using standard functional and anatomical analysis methods. In the case of anatomical images, we may utilize automated programs for parcellating brain regions, measuring cortical thickness and curvature, and specialized algorithms for specifying white matter track direction. Analysis of functional MRI data will follow the basic procedure of 1) image registration both to other functional images and the associated anatomical images, 2) conversion to standardized brain space, 3) intensity standardization followed by 4) individual inferential statistical tests such as multiple regression using convolved task time series as predictor variables. Group analyses of functional data will include both parametric and non-parametric statistical methods in standard image space in order to determine the reliability of within-group effects as well as to determine the presence of between-group differences. During data processing, analysts will remain blinded to group membership whenever possible. Between-group power analyses of fMRI data have shown that acceptable effect sizes can be obtained with 16 to 20 subjects per group. These effect sizes are significantly influenced both by voxel size, type of task, and the brain region of interest. Calculation of sample sizes will follow the statistical power analysis calculation published by [29].

F. Gender/Minority/Pediatric Inclusion for Research

Women and minorities will be included in the study without prejudice according to their representation in the study population. Adult subjects will be recruited from the greater Metro Tulsa area and should thus share the racial and ethnic composition of this area. All efforts will be made to ensure that our participant population closely resembles the ethnic and racial composition of the greater Tulsa area.

G. Human Participants

We aim to recruit human subjects with the following group specific inclusion demographics over 5 years:

- 50 females with anorexia nervosa, age 18 – 40. Predominantly female recruitment is expected due to the 10:1 female to male prevalence in AN.
- 50 females with generalized anxiety disorder, age 18 – 40. Predominantly female recruitment is expected (GAD prevalence ratios are 2:1 female to male) and in order to facilitate group matching.
- 150 healthy participants, males and females, ages 18 – 80. Mostly female participants ages 18 – 40 will be recruited to match to the psychiatric (AN, PD, GAD, MDD) samples.
-

Inclusion criteria (all participants):

(1) Must have a body mass index between 17 to 35 kg/m²
(2) Must be able to provide written informed consent and must have sufficient proficiency in the English language to understand and complete interviews, questionnaires, and all other study procedures.
(3) Must be capable of performing all tasks during each session of the experiment.

Inclusion criteria (AN participants, n = 50):
Participants (ages 18 to 40) must meet DSM 5 criteria for Anorexia Nervosa, either current or lifetime, or have an Eating Disorder Screen (SCOFF) score ≥ 2 and a current

BMI of 17 or greater. Selected medications are allowed, including selective serotonin reuptake inhibitors and benzodiazepines. Mood stabilizers and antipsychotic medications are excluded.

Inclusion criteria (GAD participants, n = 50):
Participants (ages 18 to 40) must meet DSM 5 criteria for GAD, either current or lifetime, or have an Overall Anxiety Severity and Impairment Scale (OASIS) ≥ 8 or a Generalized Anxiety Disorder 7 (GAD 7) score > 10. Selected medications are allowed, including selective serotonin reuptake inhibitors and benzodiazepines. Mood stabilizers and antipsychotic medications are excluded.

Inclusion criteria (healthy participants, n = 150):
Participants (ages 18 to 80) will be selected who have not met criteria for any DSM 5 psychiatric disorder or have current scores in the non-clinical ranges on the PHQ 9, GAD 7, OASIS, HDRS or SCOFF.

Exclusion criteria (all participants):
1) No telephone or limited access to a telephone
2) Has any of the following DSM 5 disorders:
    a. Schizophrenia Spectrum and Other Psychotic Disorders
    b. Bipolar and Related Disorders
    c. Antisocial Personality Disorder
3) Active suicidal ideation with intent or plan
4) Obesity with a body mass index > 35 preventing scanner entry.
5) Illicit stimulant drugs consumed within the past week including methamphetamine or cocaine, assessed via urine drug screen
6) Active drug or alcohol dependence, or active binge drinking within the last month
7) Pregnancy as detected by a urine test
8) Prescription of a medication outside of the accepted range, as determined by best clinical practices and current research.
9) Change in the dose or prescription of a medication within the 6 weeks before enrolling in the study that could affect brain functioning, e.g., anxiolytics or antidepressants.
10) Taking drugs that affect the fMRI hemodynamic response (e.g., methylphenidate, acetazolamide, excessive caffeine intake > 1000 mg/day).
11) Presence of unstable cardiac, vascular, pulmonary, gastrointestinal, endocrine, neurologic, hematologic, rheumatologic, or metabolic disease; or any other condition that, in the opinion of the investigator, would make participation not be in the best interest (e.g., compromise the well-being) of the subject or that could prevent, limit, or confound the protocol-specified assessments.
12) Non-correctable vision or hearing problems.
13) Systolic blood pressure > 160 mmHg
14) Diastolic blood pressure > 100 mmHg

Additional exclusion criteria (AN participants):
1) Any AN individual reporting a history of cardiac or respiratory disease
2) AN with comorbid PD will be excluded, as enhanced IA has been previously hypothesized in PD and could therefore confound the results.
3) AN with 12-lead EKG abnormalities other than bradycardia or occasional

premature ventricular complexes (PVCs); those with severe bradycardia, e.g. HR less than 40 bpm will be excluded.

4) AN reporting a seizure within the past year
5) Active antipsychotic, lithium, stimulant, or wellbutrin medication prescription
6) MRI contraindications including: cardiac pacemaker, metal fragments in eyes/skin/body (shrapnel), aortic/aneurysm clips, prosthesis, by-pass surgery/coronary artery clips, hearing aid, heart valve replacement, shunt (ventricular or spinal), electrodes, metal plates/pins/screws/ wires, or neuro/bio-stimulators (TENS unit), persons who have ever been a professional metal worker/welder, history of eye surgery/eyes washed out because of metal, vision problems uncorrectable with lenses, inability to lie still on one's back for 60-120 minutes; prior neurosurgery; tattoos or cosmetic makeup with metal dyes, unwillingness to remove body piercings, and pregnancy.

Additional exclusion criteria (GAD patients)

1) Any GAD patient reporting a history of cardiac or respiratory disease
2) GAD with comorbid PD or AN will be excluded, as altered IA has been previously hypothesized in PD and could therefore confound the results.
3) GAD with 12-lead EKG abnormalities other than bradycardia or occasional premature ventricular complexes (PVCs); those with severe bradycardia, e.g. HR less than 40 bpm will be excluded.
4) GAD reporting a seizure within the past year
5) Active antipsychotic, lithium, stimulant, or wellbutrin medication prescription
6) MRI contraindications including: cardiac pacemaker, metal fragments in eyes/skin/body (shrapnel), aortic/aneurysm clips, prosthesis, by-pass surgery/coronary artery clips, hearing aid, heart valve replacement, shunt (ventricular or spinal), electrodes, metal plates/pins/screws/ wires, or neuro/bio-stimulators (TENS unit), persons who have ever been a professional metal worker/welder, history of eye surgery/eyes washed out because of metal, vision problems uncorrectable with lenses, inability to lie still on one's back for 60-120 minutes; prior neurosurgery; tattoos or cosmetic makeup with metal dyes, unwillingness to remove body piercings, and pregnancy.

Additional exclusion criteria (healthy participants)

1) Any HC individual reporting a history of cardiac or respiratory disease
2) Any HC with 12-lead EKG abnormalities other than occasional PVCs.
3) Any HC reporting a seizure within the past year
4) MRI contraindications including: cardiac pacemaker, metal fragments in eyes/skin/body (shrapnel), aortic/aneurysm clips, prosthesis, by-pass surgery/coronary artery clips, hearing aid, heart valve replacement, shunt (ventricular or spinal), electrodes, metal plates/pins/screws/ wires, or neuro/bio-stimulators (TENS unit), persons who have ever been a professional metal worker/welder, history of eye surgery/eyes washed out because of metal, vision problems uncorrectable with lenses, inability to lie still on one's back for 60-120 minutes; prior neurosurgery; tattoos or cosmetic makeup with metal dyes, unwillingness to remove body piercings, and pregnancy.

Plans for recruitment and consent procedures to be followed:

Recruitment

Participants will be recruited through a pre-approved LIBR Screening protocol (WIRB # 20101611), through the LIBR database, through radio and internet advertisements, and through word of mouth via cardiologists in the community. Participants who meet our inclusion/exclusion criteria will be offered the opportunity to participate.

Consenting will be conducted in private exam rooms at the Laureate Institute for Brain Research. Consent will be obtained by members of the research team that have received training on the consenting process for this study. Family members will be allowed to be present and discuss the consenting process with the participant if requested. Written informed consent will be obtained from each participant after they have been provided a full verbal and written explanation of the study purpose, procedures, risks and benefits, and after they have been allowed sufficient opportunity to review this information and ask questions concerning any aspect of the study.

MRI screening procedures

For MRI studies, each participant will complete an MR safety-screening questionnaire immediately upon completion of the consent form. The MRI screening questionnaire is developed and distributed by the Institute for Magnetic Resonance Safety, Education, and Research (IMRSER) in Los Angeles, CA[1]. IMRSER is a non-profit organization sponsored by major MRI-related corporations, including GE (the manufacturer of LIBR's 3T magnet), to "disseminate information regarding current and emerging MR safety issues" and "to develop and provide materials and resources to facilitate MR safety-related education and training". The safety-screening questionnaire probes for possible occupational exposure to metal slivers or shavings (remnants of which may remain lodged in a subject's head or neck), surgical clips or shrapnel, cochlear implants, or any other form of ferrous metal implanted in or on the participant's body. Participants answering in the affirmative to any of these conditions will be excluded. All participants with any form of implanted wires, metal, or electronic devices will be excluded. Although there are no known risks of MR to pregnant women, there may be unknown risks. Therefore, females who are pregnant must not participate in this protocol.

Compensation for time spent participating

Participants in both interventions (pharmacological and breathing) will be compensated at a flat rate of $50 for completing the screening visit (e.g., collection of demographic and psychometric variables, psychiatric and medical history assessment, 12-lead EKG screening, urine samples, verification of MRI eligibility).

Pharmacological intervention: participants will receive a total of $150 per visit, upon completion of the visit. Participants will receive an additional $50 per hour of MRI scanning, prorated to the nearest half hour. Thus the maximum study compensation for two infusion visits with 2 hours of MRI scanning during each visit will be $550. Participants will be compensated $20 for each remote follow up visit, which will be

[1] INSTITUTE FOR MAGNETIC RESONANCE SAFETY, EDUCATION, AND RESEARCH 7511 McConnell Avenue, Suite 100, Los Angeles, CA 90045
http://www.imrser.org

approximately 1 hour each.

Participants will be paid with a ClinCard (a pre-paid MasterCard designed for clinical research payments). The ClinCard will be given to the participant at the end of each study visit and funds are available within about 24hrs.

H. Risks

Risks associated with self-report questionnaires:

The risks associated with completing the self-report questionnaires are minimal. Some of the questions may be uncomfortable to answer and long questionnaires may elicit boredom.

Risks associated with clinical diagnosis:

Participants may learn of their clinical diagnosis for the first time during the participation in the research. This may be discomforting to some individuals and may evoke maladaptic coping responses such as anger and denial. The likelihood of this occurrence is low, as participants are usually aware of their diagnosis or clinical symptoms.

Risks associated with the psychophysiological measures:

Measurements of autonomic nervous system activity will be made throughout the experiment. These measurements are made with electrodes placed on the surface of the skin, which can be expected to cause about the same discomfort as might be experienced with wearing an adhesive bandage. The breath hold and cold pressor tasks are measures of distress tolerance and autonomic functioning and consequently they may induce states of acute pain and/or frustration that quickly resolve as soon as the challenge is over. Additionally, the participant may experience lightheadedness from trying to hold their breath, and cold or numbness in their hand and fingers from placing their hand in very cold water. The heartbeat tapping task might induce some frustration for participants who are unable to feel their heartbeat.

Risks associated with intravenous line:

The procedure for intravenous line placement is the same as that used for routine clinical care. Insertion of the needle may be painful for a brief time. The level of risk is considered to be minimal. The risks of intravenous line placement are minimal. Possible mild side effects include bruising at the site of the IV. The risks for placement of an IVP include local bleeding, possible infection, or local inflammation of the skin and/or vein with pain and swelling. During all IV line placement, a physician or nurse will utilize sterile techniques.

Risks associated with MRI:

MRI uses powerful magnetic fields and weak radio frequency pulses (electromagnetic radiation), neither of which has been associated with adverse effects in patients or laboratory animals when studied under clinical imaging protocols. MRI centers across the country are regularly using up to 4 Tesla MRI scanners for research purposes.

However, there are the following sources of risk: (1) the participant may experience physical discomfort being in the scanner, (2) some participants may feel anxious or claustrophobic in the confined space of the MRI scanner, (3) the strong magnetic field will affect electronic, magnetic, and metal devices that participants carry with them or that have been implanted in the participant's body, (4) occasionally some participants may experience muscle twitching or paresthesias, especially in the torso, due to peripheral nerve stimulation effects of the MRI scanner, (5) some individuals may experience light-headedness or dizziness while in the scanner or when rising from the MRI gurney too rapidly, and (6) MRI scanners produce a loud high frequency tone that can cause hearing damage if appropriate hearing protection is not used.

Risks associated with isoproterenol:

It is possible that participants may have an adverse (allergic) reaction to isoproterenol. It is possible that during the isoproterenol infusions some individuals may experience anxiety, tremors, flushing, palpitations, dyspnea, headache, hypotension, premature ventricular contraction or nausea. The frequency of these side effects is not well documented, although they have been shown to occur with increasing frequency at high doses (e.g. at heart rates greater than 160 bpm). Finally, it is possible that participating in this task may be physically tiring.

Minimizing risks associated with self report questionnaires:

In order to minimize risks associated with the self-report measures, participants will be informed that they may decline to answer any specific questions, or discontinue the interview at any time, that their participation is strictly voluntary and they have the right to withdraw at any time without penalty.

Minimizing risks associated with clinical diagnosis:

In order to minimize the risk to participants, when first making the diagnosis a licensed nurse or physician study member will provide education about the disorder including the prevalence, typical symptoms and comorbid diagnoses, course, treatment options, and prognosis. The participant will be given the opportunity to ask questions of the nurse or physician study member about any aspects of their diagnosis, and will be provided with contact phone numbers if they think of additional questions at a later time. They will also be given referrals to treatment providers in their area who can help them.

Minimizing risks associated with psychophysiological testing:

Participants are free to stop the breathhold and cold pressor tasks at anytime, which will immediately remove any feelings of distress. Moreover, all of the tasks are short, which should minimize levels of frustration, lightheadedness, and cold/numbness.

Minimizing risks associated with intravenous line:

In order to minimize risks associated with the intravenous line, the trained phlebotomist/nurse will always utilize sterile techniques and provide a bandage upon removal.

Minimizing risks associated with isoproterenol:

In order to minimize risks associated with isoproterenol, a careful medical history, physical exam is taken to detect the presence of medical conditions which would preclude study participation. Subjects screening positive for abnormal physical exam findings (e.g. systolic ejection murmur, diastolic murmur) will be excluded. Additionally, a 12 lead EKG is recorded and reviewed by a board certified cardiologist. Any participant for whom the cardiologist recommends against study participation will be excluded. We will also measure urine pregnancy and urine drug screen and will exclude any female participant found to be pregnant or any participant found to have stimulants (cocaine, amphetamines), to prevent potential adverse blood pressure elevations. Participants will be screened for a history of an allergic reaction to isoproterenol, and will be given a brief test with a very low dose (0.1 mcg) before starting the experiment to ensure tolerance. Participants will be monitored at each step of the experiment; they will be reminded that they can discontinue the experiment at any time, and each portion of the experiment will only proceed after obtaining the subject's permission. Mental fatigue will be monitored closely and subjects will be provided appropriate rest breaks. During the isoproterenol infusion portion of the study, if a participant experiences emotional distress that is more than mild to moderate in severity and/or is not short-lived, a licensed study physician will be immediately available to meet with them. Participants will be screened for a resting heart rate below 100 bpm to ensure that at the highest level of isoproterenol induced arousal the subject's heart rate is below 160 bpm. During the experiment participants will be continuously monitored via electrocardiogram or pulse oximeter and respirometer, to monitor changes induced by the intervention. Blood pressure will be periodically measured throughout the experiment. Additionally, participants will be asked at regular intervals how they are feeling, and whether they wish to continue with the study. The isoproterenol infusion will be terminated if any ECG abnormalities or heart rate elevations > 160 bpm are observed during the procedure. Finally, to alleviate physical fatigue, subjects will be positioned in a cushioned chair and will be provided with frequent ambulatory breaks.

Minimizing risks associated with MRI:

In order to minimize risks associated with MRI, each participant will complete a safety-screening questionnaire prior to entering the MR environment. The safety pre-screening questionnaire probes subjects for possible occupational exposure to metal slivers or shavings remnants of which may remain lodged in the subject’s head or neck. Participants with surgical clips or shrapnel, cochlear implants, or any other form of ferrous metal body implanted in or on their body will be excluded. All participants with any form of implant wires, metal or electronic device implants will be excluded. All persons involved in this protocol will receive MR safety training conducted at the Laureate Institute for Brain Research by their MR safety officer.

We intend to minimize claustrophobic problems using a series of procedures: 1) by giving a detailed explanation of the environment prior to scanning, 2) maintaining voice contact with the subjects at all times, 3) maintaining visual contact of the participant in the scanner using observational cameras placed inside the scanner room. Participants will also be provided with the opportunity to lay in a mock scanner prior to the actual MRI.

To minimize the risk of hearing damage, all participants will be fitted with ear plugs and be required to wear the hearing protection for the duration of the MRI scanning.

Potential benefits and importance to the participants and others:

Participants in the pharmacological intervention will not experience any direct benefits from participation. The results of these studies will further the scientific community's understanding of eating, anxiety and mood disorder pathophysiology and may ultimately lead to better treatments. As such, participants may derive personal satisfaction from their contribution to the discovery process.

Risk/benefit analysis:

The risks for the pharmacological intervention are greater than minimal with the research and there are no direct benefits to the subjects. However, the risk/benefit ratio is favorable when considering the results of the research may benefit the ability of future studies to provide better treatments for individuals with eating disorders, anxiety disorders or depression, conditions for which current state of the art treatments fail a majority of the time. Study participation is not expected to prevent or delay treatment, and all participants with eating, anxiety or mood disorders will be encouraged to seek treatment after the study or if they elect not to participate.

Plan of Action for Incidental Findings:

Upon detection of incidental findings during MRI scanning or during physiological recording (e.g., ECG), an investigator (study nurse or physician) will communicate the discovery to the participant verbally and/or by written communication. The participant will be encouraged to contact their primary-care physician about the discovery. LIBR will provide a digital copy of the suspect MR scans and/or physiological data to the primary-care physician upon request once the participant provides written consent authorizing the release of these medical records. Additionally, detection and disclosure of incidental findings will be documented in a database contained on the Laureate Institute for Brain Research computer cluster.

Emergency protocol:

In the instance of an adverse event (e.g., clinically significant hypotension, fainting), the following standard CTRC procedures will be followed:

1. Infusion protocol will be stopped immediately

2. For hypotension, the patient will be placed in the trendelenburg position to quickly enhance blood pressure and cerebral perfusion.

3a. If this procedure achieves resolution of hypotension or return of consciousness, the patient will be excused from the study

3b. If this procedure does not result in resolution of hypotension or return of consciousness, then the remainder of the emergency protocol will be instituted (see #4 below). Similarly, the following procedures will also be employed if the patient is found to be in asystole, or is pulseless and unresponsive, or reports any other potentially adverse outcome (e.g. chest pain).

4. An Advanced Cardiovascular Life Support (ACLS) certified member of the study nursing staff will immediately begin performing cardiopulmonary resuscitation (CPR). They will rotate CPR with other Basic Life Support (BLS) certified members of the study staff including the study physician (MD).

5. Simultaneously, a member of the study staff will call for emergency response services by dialing 911. Based on prior experience, the response time is approximately 5 minutes for the Emergency Medical Services Authority (EMSA).

6. Emergency medical services will determine the need for implementing the appropriate ACLS protocol.

7. Emergency medical services responders will determine the need for transfer to the Saint Francis Healthcare System Emergency Department (located across the street) for further evaluation and management.

I. Data Safety and Monitoring Board

A Data Safety Monitoring Board (DSMB) has been convened. The DSMB will meet no less than twice per year. Adverse events and deviations will be made known to the Laureate Institute for Brain Research Human Protection Administrator at (918) 502-5155 or via email at hpa@laureateinstitute.org. All reportable adverse events and/or deviations will be disclosed to the DSMB and to the Western IRB.

J. Confidentiality

To protect participant confidentiality, participant data will be anonymized as follows:

1. Last name: All participants will be assigned the last name “LIBR.”

2. First name: The first name will be a secure alpha cryptographic hash based on LIBR participant ID. This technique is the gold standard in computer security for one-way correlation of data.

Records of the participant’s participation in this study will be held confidential, except when disclosure is required by law, or as described in the informed consent document (under "Confidentiality"). The study doctor, the sponsor or persons working on behalf of the sponsor, and under certain circumstances, the United States Food and Drug Administration (FDA) and the Institutional Review Board (IRB) will be able to inspect and copy confidential study-related records that identify the subject by name. Therefore, absolute confidentiality cannot be guaranteed. If the results of this study are published or presented at meetings, the subject will not be identified.

Subjects in MR studies will have both anatomical and functional MRI scans. Paper copies of consents, screening forms, the Research Privacy Form, and any other forms, testing results or papers containing Personally Identifiable Information (PII) will be stored in a secured room with access granted only to authorized personnel. Data will be stored electronically on an encrypted, password protected network.

There is risk of possible loss of participant confidentiality. To minimize risk, all study records that identify the participant (including contact information such as phone number and email address) will be kept in a locked record room and only study personnel will have the entry code. All hard copy data will be stored in a locked cabinet. Any study records entered into a computer system will be assigned code numbers and will not be individually identifiable. Code numbers are a combination of numbers and letters.

References:

1. Craske, M.G., K. Kircanski, A. Epstein, H.-U. Wittchen, D.S. Pine, R. Lewis-Fernández, and D. Hinton, Panic disorder: a review of DSM-IV panic disorder and proposals for DSM-V. Depression and Anxiety, 2010. 27(2): p. 93-112.
2. Weisberg, R.B., Overview of generalized anxiety disorder: epidemiology, presentation, and course. Journal of Clinical Psychiatry, 2009. 70(2): p. 4-9.
3. Halmi, K.A. and S.R. Sunday, Temporal patterns of hunger and fullness ratings and related cognitions in anorexia and bulimia. Appetite, 1991. 16(3): p. 219-37.
4. Strober, M., R. Freeman, C. Lampert, and J. Diamond, The association of anxiety disorders and obsessive compulsive personality disorder with anorexia nervosa: Evidence from a family study with discussion of nosological and neurodevelopmental implications. International Journal of Eating Disorders, 2007. 40(S3): p. S46-S51.
5. Waller, G., J. Evans, and M. Pugh, Food for thought: a pilot study of the pros and cons of changing eating patterns within cognitive-behavioural therapy for the eating disorders. Behavior Research & Therapy, 2013. 51(9): p. 519-25.
6. Dunn, B.D., T. Dalgleish, A.D. Ogilvie, and A.D. Lawrence, Heartbeat perception in depression. Behav Res Ther, 2007. 45(8): p. 1921-30.
7. Dunn, B.D., I. Stefanovitch, D. Evans, C. Oliver, A. Hawkins, and T. Dalgleish, Can you feel the beat? Interoceptive awareness is an interactive function of anxiety- and depression-specific symptom dimensions. Behav Res Ther, 2010. 48(11): p. 1133-8.
8. Pollatos, O., E. Traut-Mattausch, and R. Schandry, Differential effects of anxiety and depression on interoceptive accuracy. Depression and Anxiety, 2009. 26(2): p. 167-173.
9. Avery, J.A., W.C. Drevets, S.E. Moseman, J. Bodurka, J.C. Barcalow, and W.K. Simmons, Major depressive disorder is associated with abnormal interoceptive activity and functional connectivity in the insula. Biological Psychiatry, 2014. 76(3): p. 258-66.
10. Khalsa, S.S., D. Rudrauf, C. Sandesara, B. Olshansky, and D. Tranel, Bolus isoproterenol infusions provide a reliable method for assessing interoceptive awareness. Int J Psychophysiol, 2009. 72(1): p. 34-45.

11. Khalsa, S.S., D. Rudrauf, and D. Tranel, Interoceptive awareness declines with age. Psychophysiology, 2009. 46(6): p. 1130-1136.

12. Jones, G.E., Perception of visceral sensations: a review of recent findings, methodologies, and future directions. Advances in Psychopysiology, , ed. J.R. Jennings, Ackels, P.K. Vol. 5. 1994, London: Jessica Kingsley Publishers.

13. Khalsa, S.S., D. Rudrauf, A.R. Damasio, R.J. Davidson, A. Lutz, and D. Tranel, Interoceptive awareness in experienced meditators. Psychophysiology, 2008. 45(4): p. 671-77.

14. Ring, C. and J. Brener, Influence of beliefs about heart rate and actual heart rate on heartbeat counting. Psychophysiology., 1996. 33(5): p. 541-6.

15. Ring, C., J. Brener, K. Knapp, and J. Mailloux, Effects of heartbeat feedback on beliefs about heart rate and heartbeat counting: a cautionary tale about interoceptive awareness. Biological Psychology, 2015. 104: p. 193-8.

16. Windmann, S., O.W. Schonecke, G. Frohlig, and G. Maldener, Dissociating beliefs about heart rates and actual heart rates in patients with cardiac pacemakers. Psychophysiology, 1999. 36(3): p. 339-42.

17. George, C.F., M.E. Conolly, T. Fenyvesi, R. Briant, and C.T. Dollery, Intravenously administered isoproterenol sulfate dose-response curves in man. Arch Intern Med, 1972. 130(3): p. 361-4.

18. Cleaveland, C.R., R.E. Rangno, and D.G. Shand, A standardized isoproterenol sensitivity test. The effects of sinus arrhythmia, atropine, and propranolol. Arch Intern Med, 1972. 130(1): p. 47-52.

19. Contrada, R.J., J. Dimsdale, L. Levy, and T. Weiss, Effects of isoproterenol on T-wave amplitude and heart rate: a dose-response study. Psychophysiology, 1991. 28(4): p. 458-62.

20. Mills, P.J., J.E. Dimsdale, S. Ancoli-Israel, J. Clausen, and J.S. Loredo, The effects of hypoxia and sleep apnea on isoproterenol sensitivity. Sleep, 1998. 21(7): p. 731-5.

21. Khalsa, S.S., M.G. Craske, W. Li, S. Vangala, M. Strober, and J.D. Feusner, Altered interoceptive awareness in anorexia nervosa: Effects of meal anticipation, consumption and bodily arousal. LID - 10.1002/eat.22387 [doi]. International Journal of Eating Disorders, 2015. doi: 10.1002/eat.22387 [Epub ahead of print].

22. Asmundson, G.J. and M.B. Stein, Triggering the false suffocation alarm in panic disorder patients by using a voluntary breath-holding procedure. American Journal of Psychiatry, 1994. 151(2): p. 264-6.

23. Edes, B.D. and K.M. Dallenbach, The adaptation of pain aroused by cold. The American Journal of Psychology, 1936. 48: p. 307-15.

24. Lovallo, W.R., The cold pressor test and autonomic function: a review and integration. . Psychophysiology, 1975. 12(3): p. 268-82.

25. Arnold, J.M. and D.G. McDevitt, Standardised isoprenaline sensitivity tests--a comparison of existent methods. Br J Clin Pharmacol, 1983. 15(2): p. 167-72.

26. Kaye, W.H., D.T. George, H.E. Gwirtsman, D.C. Jimerson, D.S. Goldstein, M.H. Ebert, and C.R. Lake, Isoproterenol infusion test in anorexia nervosa:

assessment of pre- and post-beta-noradrenergic receptor activity. Psychopharmacol Bull, 1990. 26(3): p. 355-9.

27. Avery, J., K. Kerr, K. Burrows, J.C. Barcalow, J. Bodurka, and W.K. Simmons, Activity within the mid-insula primary interoceptive cortex is related to the conscious experience of interoceptive signals from the heart. Society for Neuroscience Abstract Viewer and Itinerary Planner, 2013. 43.

28. Mulert, C., G. Leicht, P. Hepp, V. Kirsch, S. Karch, O. Pogarell, M. Reiser, U. Hegerl, L. Jager, H.J. Moller, and R.W. McCarley, Single-trial coupling of the gamma-band response and the corresponding BOLD signal. Neuroimage, 2010. 49(3): p. 2238-47.

29. Desmond, J.E. and G.H. Glover, Estimating sample size in functional MRI (fMRI) neuroimaging studies: statistical power analyses. Journal of Neuroscience Methods, 2002. 118(2): p. 115-28.
